# Membrane transporters in cell physiology, cancer metabolism and drug response

**DOI:** 10.1242/dmm.050404

**Published:** 2023-12-01

**Authors:** Sara Alam, Emily Doherty, Paula Ortega-Prieto, Julia Arizanova, Louise Fets

**Affiliations:** Drug Transport and Tumour Metabolism Lab, MRC Laboratory of Medical Sciences, Hammersmith Hospital Campus, Du Cane Road, London, W12 0NN, UK

**Keywords:** Cancer Metabolism, Drug Uptake, Pharmacology, Transporters

## Abstract

By controlling the passage of small molecules across lipid bilayers, membrane transporters influence not only the uptake and efflux of nutrients, but also the metabolic state of the cell. With more than 450 members, the Solute Carriers (SLCs) are the largest transporter super-family, clustering into families with different substrate specificities and regulatory properties. Cells of different types are, therefore, able to tailor their transporter expression signatures depending on their metabolic requirements, and the physiological importance of these proteins is illustrated by their mis-regulation in a number of disease states. In cancer, transporter expression is heterogeneous, and the SLC family has been shown to facilitate the accumulation of biomass, influence redox homeostasis, and also mediate metabolic crosstalk with other cell types within the tumour microenvironment. This Review explores the roles of membrane transporters in physiological and malignant settings, and how these roles can affect drug response, through either indirect modulation of sensitivity or the direct transport of small-molecule therapeutic compounds into cells.

## Teaser

**Summary:** This Review examines the function of SLC family membrane transporters in physiology and disease, with a focus on cancer metabolism and how this intersects with their role in drug uptake.

## Introduction

Plasma and organellar membrane transporters play a vital role in the import and export of a diverse range of endogenous and exogenous small molecules and ions, making them essential for whole-body, cell and organellar homeostasis. Given their importance for tissue and cell homeostasis, it's unsurprising that membrane transporters also play important roles in a variety of diseases, from rare inborn errors of metabolism to neurodegenerative disorders and, perhaps most notably, across a broad spectrum of cancer types.

In the context of cancer, changes in membrane transporter expression can be driven by a range of oncogenic mutations. These expression changes facilitate cell-intrinsic, pro-proliferative metabolic alterations and adaptations to, as well as metabolic crosstalk with, the tumour microenvironment. Several superfamilies of transporters exist, classed broadly by their mechanism of transport. The largest and most diverse of these is the Solute Carrier (SLC) transporter superfamily, comprising over 450 members that transport ions and metals, sugars, amino acids, nucleosides, and many other solutes across membranes (Meixner et al., 2020; Tweedie et al., 2020; Ferrada and Superti-Furga, 2022). The physiological function of SLCs, as well as their roles in cancer cell metabolism and how this may impact response to drugs, is the main focus of this Review. For consistency, transporters will be referred to by their SLC name, with any additional common nomenclature in brackets. Further information on the SLC nomenclature system has been reviewed by Hediger et al. (2013).

## Structure, function and physiological roles of membrane transporters

**Fig. 1.**
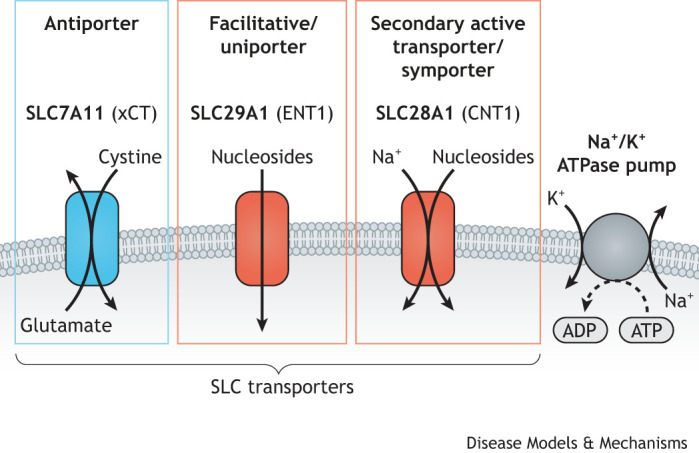
**Types of SLC-mediated transport.** Examples of plasma membrane SLC transporter types, showing their substrates and mechanisms of transport. The Na^+^/K^+^ ATPase pump (right) is an example of an active transporter that generates a Na^+^ gradient across the plasma membrane. This gradient drives the movement of Na^+^ ions and nucleosides via SLC28A1, making this SLC a secondary active transporter. SLC29A1, by contrast, is a facilitative uniporter that enables passage of nucleosides across the membrane dependent on their concentration gradient. SLC7A11 is an example of an antiporter and uses export of glutamate to drive the influx of cystine, the oxidised form of cysteine. The transporters are colour-coded based on their main substrate (see legend to Fig. 2 for details).

Structurally, SLC transporters are a highly diverse group of multi-membrane pass proteins, with the Leu-T and the Major Facilitator Superfamily (MFS) comprising the two most common structural folds. Significant advances in membrane protein structural biology made over the past decade have hugely improved our understanding of how transport takes place at the molecular level – with mechanisms including Rocker Switch, Gated-Pore and Elevator, that have been reviewed elsewhere (Drew and Boudker, 2015; Colas et al., 2016; Dvorak and Superti-Furga, 2023). Functionally, SLCs are classed as either facilitative or secondary active transporters. Facilitative transporters allow the passage of a substrate down its concentration gradient, while secondary active transporters translocate substrates by utilising the electrochemical gradient of a second substrate, usually an ion, enabling the movement of the primary substrate against its concentration gradient (Crane, 1960) (Fig. 1). Co-transported substrates can either be translocated in the same (symporter) or opposing (antiporter) directions. Unlike active ATP-Binding Cassette (ABC) transporters, secondary active transporters indirectly rely on the energy status of the cell by utilising ion gradients established by ATP-hydrolysing pumps (Hediger et al., 2004) (Fig. 1). Examples are members of the sodium (Na^+^)-glucose co-transporter (SGLT) SLC5 family, which drive glucose import using the Na^+^ concentration gradient generated by ion pumps/transporters, such as the Na^+^/K^+^ ATPase (Crane, 1960; Wright et al., 2011).

**Fig. 2.**
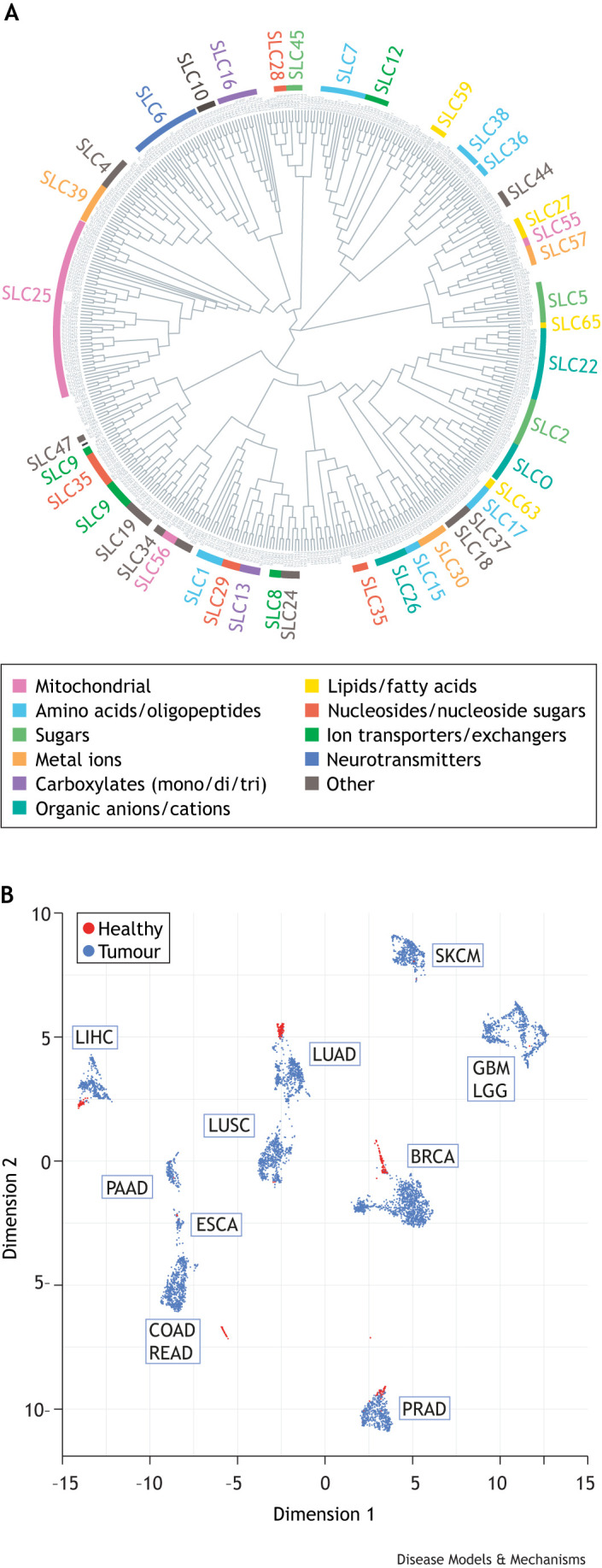
**Genetic diversity of human SLCs and transcriptional heterogeneity of transporters in cancer.** (A) A list of the SLC superfamily members was obtained from the HGNC website (https://www.genenames.org/data/genegroup/#!/group/752). As of December 2022, the superfamily contains 433 SLC transporters. Reviewed sequences were aligned using ClustalW in the R package msa, and the alignment was converted to a tree and visualised using the R packages ape and ggtree, respectively. Transporter families have been colour-coded according to substrate class, except for the mitochondrial transporters that, for evolutionary reasons, cluster together despite differential substrate preferences. (B) Dimensionality reduction analysis (using the UMAP package in R, with default settings) of patient samples from the TCGA database, based on the expression of SLC and ABC transporters. The transporter expression signatures in tumours are far more diverse (samples in blue, more dispersed) than in their matched normal tissue controls (samples in red, tightly clustered) across cancer types. This illustrates the heterogeneous expression of transporters in cancer. BRCA, breast-invasive carcinoma; COAD, colon adenocarcinoma; ESCA, oesophageal carcinoma; GBM, glioblastoma multiforme; LGG, brain low-grade glioma; LIHC, liver hepatocellular carcinoma; LUAD, lung adenocarcinoma; LUSC, lung squamous cell carcinoma; PAAD, pancreatic adenocarcinoma; PRAD, prostate adenocarcinoma; READ, rectum adenocarcinoma; SKCM, skin cutaneous melanoma.

### SLCs play a fundamental role in metabolism and physiology

The breadth of substrates makes the SLC family fundamental to many aspects of physiology and, as a result, implicates them in a number of inborn errors of metabolism (Box 1), with over 80 associated with monogenic disorders (Lin et al., 2015). The importance of SLCs is exemplified by SLC25A42, one of two known mitochondrial coenzyme A (CoA) transporters (Fiermonte et al., 2009). While synthesised in the cytoplasm (Martinez et al., 2014), CoA is required in the mitochondria for the formation of acetyl-CoA from pyruvate (Fiermonte et al., 2009), to fuel the tricarboxylic acid (TCA) cycle (Box 1) and energy metabolism, as well as for beta oxidation of fatty acids (Box 1). The movement of CoA across the inner mitochondrial membrane is, therefore, essential for fundamental metabolic functions. Indeed, children born with mutations in *SLC25A42* display a wide array of symptoms, such as mitochondrial myopathy, developmental regression and epilepsy (Shamseldin et al., 2016; Almannai et al., 2018). Other non-silencing mutations in or around the gene appear to impact lipid metabolism. In fact, single-nucleotide polymorphisms near this gene have been associated with hypertension in some studies (Fox et al., 2011), likely due to the role of CoA in beta oxidation in the mitochondrion. Interestingly, SLC25A42 expression has been shown to be upregulated in foetuses undergoing transcriptional reprogramming to adapt to placental insufficiency (Box 1) (Chou and Wang, 2020), suggesting that SLC25A42 upregulation may contribute to the metabolic reprogramming required for foetal survival in sub-optimal nutritional environments.

**Box 1. Glossary**

**Allosteric:** the binding of a small molecule to a protein in a pocket outside of the active binding site or the substrate-binding site.

**Anaplerosis:** ‘topping up’ of TCA cycle intermediates with carbon sources, such as pyruvate, glutamate or fatty acids.

**Beta oxidation:** the breakdown of fatty acids to acetyl-CoA, which can subsequently enter the TCA cycle to release energy in the form of ATP.

**Caco-2 cell line:** an immortalised human colorectal cancer cell line that is often grown to a monolayer to mimic intestinal epithelial barrier for use in transport assays.

**Creatine:** a metabolite that can be reversibly phosphorylated by creatine kinase enzymes to form phospho-creatine. Dephosphorylation of phospho-creatine is coupled to the formation of ATP and, as such, can act as an energy shuttle to ‘transport’ ATP to specific subcellular sites.

**DMOG/MOG (dimethyloxalylglycine/methyloxalylglycine):** a tool compound that has been classically used to inhibit prolyl hydroxylases and other α-ketoglutarate dependent dioxygenases. In cell culture medium and blood, DMOG is rapidly de-esterified to form MOG, which is a substrate of SLC16A7. Intracellularly, both DMOG and MOG are fully de-esterified to form the active inhibitor NOG (N-oxalylglycine), which can bind to a range of different α-ketoglutarate-dependent enzymes, depending on the intracellular concentration reached.

**Ergosterol:** a cholesterol-like hydrophobic molecule and a main sterol in yeast.

**FDG-PET (fluorodeoxyglucose–positron emission tomography) imaging:** a diagnostic imaging technique, which relies on positron emission tomography to visualise a radiolabelled glucose analogue entering tumours via the glucose transporter SLC2A1, the latter being highly upregulated in many tumours.

**Ferroptosis:** an iron-dependent form of cell death associated with redox imbalance and the accumulation of lipid peroxides, distinct from other types of cell death, such as apoptosis or necrosis.

**Gemcitabine:** a nucleoside analogue drug used in the treatment of numerous cancer types. Its mechanism(s) of action include inhibition of DNA replication.

**HIF1A (hypoxia inducible factor 1 subunit alpha):** a transcription factor that is stabilised and translocated to the nucleus in response to low oxygen tensions, instigating a transcriptional programme that allows cellular adaptation to hypoxic conditions.

**Inborn errors of metabolism:** a class of genetic disorders caused by mutations within genes encoding metabolic enzymes, including transporters.

**Lipinski's rule of five:** an approximate method traditionally used by medicinal chemists to assess the physicochemical properties of small molecules to predict oral availability in drug design.

**Metformin:** a member of the biguanide class of drugs, which is widely used in the treatment of type-II diabetes. It is also being explored as an anti-neoplastic agent.

**Orthosteric:** the binding of a molecule within a known active or substrate-binding pocket.

**PARP (poly ADP ribose polymerase) protein family:** a group of enzymes involved in DNA repair and other cellular processes. PARP1 and PARP2 bind to single-strand DNA breaks and recruit factors involved in base excision repair. Drugs that inhibit these enzymes, e.g. Olaparib, cause synthetic lethality in tumours that carry mutations in genes required for homologous recombination repair.

**Pentose phosphate pathway:** a pathway within central carbon metabolism, which diverges from glycolysis, and is important for the formation of nucleotide precursors and the generation of NADPH.

**Placental insufficiency:** a condition during pregnancy in which oxygen and nutrient supply to the foetus is disrupted.

**TCA (tri-carboxylic acid) cycle:** the TCA (also known as Krebs) cycle is a pathway within central carbon metabolism, which allows for the oxidation of acetyl-CoA derived from glucose and other carbon sources to fuel oxidative phosphorylation and subsequent ATP production.

**Syntrophy:** also known as ‘cross-feeding’, syntrophy refers to a dependence on metabolite sharing between neighbouring cooperative species or cells.

Beyond inborn errors of metabolism, several SLCs have been associated with predisposition to or increased severity of multi-factorial diseases (reviewed previously by Lin et al., 2015), such as type II diabetes (Barragán-Álvarez et al., 2021), hypertension (Boedtkjer et al., 2011; International Consortium for Blood Pressure Genome-Wide Association Studies et al., 2011), obesity (Le et al., 2021; Hodges et al., 2022), depression (Santarelli et al., 2015), cancer (Nimmanon et al., 2017; Fang et al., 2021; Han et al., 2021; Mao et al., 2021) and aging (Crocco et al., 2018). For example, at synapses and in astroglia, the SLC1 (Xu et al., 2016) and SLC6 (Kristensen et al., 2011) families of amino acid transporters are responsible for the removal of neurotransmitters from the synaptic cleft, quenching postsynaptic effects after neurotransmission, and balancing excitatory and inhibitory neurotransmitters (Aykac and Sehirli, 2020). Reduced function of the microglial glutamate transporter SLC1A2 (also known as GLT1 or EAAT2) has been associated with neurodegenerative disorders, such as Amyotrophic Lateral Sclerosis (Rothstein, 1996; Yang et al., 2009), demonstrating the vital role of SLCs within the nervous system.

### SLCs and tissue specificity

The diversity of substrate specificities and regulatory properties of SLCs allow cells to tailor transporter expression signatures to tissue- and cell type-specific metabolic requirements, as well as to their extracellular environment. Accordingly, SLCs are not only differentially but also dynamically expressed in varying cell types and tissues (Nishimura and Naito, 2008; Morioka et al., 2018; O'Hagan et al., 2018), even as early as in the developing embryo (Saunders et al., 2015; Schoels et al., 2021). Indeed, some tissue functions rely heavily on the import or export of specific metabolites or ions. For example, creatine (Box 1) is synthesised in the liver and kidneys (da Silva et al., 2009) but is mostly utilised in cell types with high energy demand, such as skeletal muscle and neuronal cells (Bessman and Geiger, 1981; Kazak and Cohen, 2020). The functioning of these cells, therefore, depends on the creatine transporter SLC6A8 (also known as CRT1), and reduced function of this transporter leads to symptoms such as intellectual disability and seizures (Cervera-Acedo et al., 2015; Tise et al., 2023).

In the kidney, the central role of filtering nutrients and salts from the blood means that SLC transporters are essential for the physiology of this organ. 380 SLCs are significantly expressed in renal tissues, with many being differentially expressed along the kidney tubules (Kanai et al., 1994; Lewis et al., 2021). Importantly, alternative transporter isoforms with varying substrate affinities may be preferentially expressed in specific locations or in response to altered diet of an organism. Indeed, in the earlier segments of the nephron, which are exposed to a higher concentration of a wide array of salts, the epithelial cells lining the tubules mostly express low-affinity and high-capacity SLCs to enable bulk removal of concentrated solutes. In the distal nephronal regions, these SLCs are replaced by higher affinity isoforms that ensure the removal of salts even at the low remaining concentrations (Giménez et al., 2002; Mount, 2014). Indeed, feeding mice a low-salt diet triggers an upregulation of NKCC2B, the low-capacity and high-affinity splice isoform of the Na^+^/K^+^/Cl^−^ co-transporter SLC12A1 (also known as NKCC2), in the renal cortex and medulla (Schiessl et al., 2013), demonstrating the importance of SLCs in adapting organ physiology to environmental conditions.

### Regulation of SLCs

The expression level, activity and subcellular localisation of SLCs, in combination with substrate concentration gradients, determine the influx rate and, subsequently, the concentration of a substrate within the cell or a subcellular compartment. As a result, SLC activity must be tightly regulated – for example in response to substrate availability or requirement – to appropriately shape the metabolic architecture a cell adopts in response to external stimuli, such as hypoxia (Wojtal et al., 2014; Gorczyca et al., 2021). Regulation of SLCs can occur at the level of transcription (reviewed previously by Zhou and Shu, 2022) or post-transcriptionally through splicing modulation (Schiessl et al., 2013) or RNAi (reviewed previously by Yi and Yu, 2022). Short-term, dynamic changes can also occur at the post-translational level, where modifications including glycosylation, phosphorylation and ubiquitylation, can impact protein stability, folding and membrane insertion, as well as directly altering transporter kinetics (Wieman et al., 2007; Czuba et al., 2018). For example, the zinc (Zn^2+^) exporter SLC30A1 (also known as ZNT1) is glycosylated and endocytosed in the absence of Zn^2+^ while, by contrast, in the presence of Zn^2+^, a decrease in glycosylation increases its stability on the plasma membrane (Nishito and Kambe, 2019). SLCs have also been shown to be regulated by direct interaction with exogenous small molecules, including environmental chemicals, such as organophosphorus pesticides, that can activate or inhibit the transport of substrates (Chedik et al., 2019) (reviewed previously by Nicklisch and Hamdoun, 2020). In addition, when SLC transporters are bound to their substrates, they can undergo conformational changes that – as in the case of the Zn^2+^ transporter SLC39A4 (ZIP4) – may promote their removal from the plasma membrane by endocytosis, allowing substrate concentrations to directly regulate transporter numbers (Shimokawa et al., 2002; Zhang et al., 2020).

### SLCs regulate metabolism

By facilitating the movement of metabolites and salts between the intra- and extracellular space, and by regulating intracellular compartmentalisation of compounds within organelles, SLCs inevitably play a fundamental and understudied role in regulating the metabolic network of the cell. According to the rules of mass action, the import of a metabolic substrate or product typically influences the direction and activity of metabolic reactions and pathways of which they are part (Song et al., 2020). Indeed, when glucose import is increased, such as by an upregulation of SLC2A1 (GLUT1) expression, hexokinase activity, as indicated by glucose-6-phosphate levels (Fujii and Beutler, 1985), and overall glycolytic activity, as shown by increased lactate levels, also increase (Fujii and Beutler, 1985; Freemerman et al., 2014; Williamson et al., 2018; Song et al., 2020).

Furthermore, SLC transporters on organellar membranes can influence the compartmentalisation of allosteric or orthosteric (Box 1) inhibitors relative to target enzymes. The metabolome is tightly regulated by both product inhibition and by inhibition or activation of enzymes and pathways by seemingly unrelated metabolites (Lindsley and Rutter, 2006; Li et al., 2010; Gerosa and Sauer, 2011; Macpherson and Anastasiou, 2017). The extent of small molecule-protein interaction has been clearly demonstrated in the ergosterol (Box 1) biosynthesis pathway in yeast, where 16 of the 21 proteins of this pathway were found to be bound to metabolites, along with a large number of protein kinases (Li et al., 2010). Indeed, it is estimated that more than 90% of enzymatic reactions in cells can be inhibited by one or more metabolites, the most impactful of which are orthophosphate, ATP, ADP and AMP (Alam et al., 2017). ATP, for example, can inhibit numerous metabolic enzymes, such as the rate-limiting glycolytic enzymes phosphofructokinase and pyruvate kinase (Larsson et al., 2000; Sander et al., 2019). These inhibitory interactions can be managed through compartmentalisation of metabolites within the cell (Zecchin et al., 2015), whereby the substrate breadth and subcellular localisation of SLC transporters enable them to shape the metabolic network by regulating the localisation of inhibitors relative to their targets (Bar-Peled and Kory, 2022). For example, ATP/ADP/AMP/P_i_ can be transported by one or more SLCs in the SLC17, SLC25, SLC29 and SLC35 transporter families (Ruprecht et al., 2014; Kunji et al., 2016; Thompson et al., 2016), many of which are localised to the mitochondrial inner membrane, allowing cells to compartmentalise energy equivalents and regulate their inhibitory properties. Phosphofructokinase can also be regulated by citrate, an intermediate of the TCA cycle. Citrate is transported across the inner mitochondrial membrane by SLC25A1, implicating this SLC in the regulation of glycolysis by connecting inhibitor and target (Nielsen, 1983).

Importantly, a large number of SLCs transport metal ions, many of which interact with and regulate ∼40% of all enzymes in cells (Andreini et al., 2009; Aulakh et al., 2022). Zn^2+^, for example, plays a role in regulating protein kinases and phosphatases (Nimmanon et al., 2017; Slepchenko et al, 2018) that function in signal transduction as well as fundamental metabolic pathways, like glycolysis (Tamaki et al., 1983; Fitzsimmons et al., 2018). Therefore, the many SLCs that can transport Zn^2+^ as well as other metal ions, such as iron, manganese and calcium, from the extracellular space or intracellular stores, play crucial roles in regulating enzymatic activity in the cell and, by extension, cellular metabolism and homeostasis.

As well as influencing metabolic reactions, SLCs can have a broader impact on cell physiology and metabolism by influencing transcriptional regulation (Hyde et al., 2007; Wieman et al., 2007; Menga et al., 2017). Zn^2+^ transporters from the SLC39 (ZIP) family have been associated with inflammation, cell movement and cell morphology owing to the regulation of Zn^2+^-dependent transcription factors, such as STATs, Snail proteins and NF-κB, when Zn^2+^ import is altered (Yamashita et al., 2004; Leung et al., 2012; Liu et al., 2013; Geng et al., 2018). In addition, purine import via SLCs promotes the interaction between the histone acetylation reader protein BRD4 and chromatin, highlighting the impact of SLC transporters on epigenetic regulation (Li et al., 2021).

### SLCs, the extracellular environment and neighbours

SLC transporters play a central role in cellular interaction with the extracellular environment, and the SLC expression signature of a cell can heavily influence *in vitro* growth rates in culture media with differing compositions, as illustrated in a recent preprint (Chidley et al., 2023 preprint). In turn, the redox and energetic status of a cell can influence the SLC expression profile to enable cells to exploit the availability of extracellular nutrients. For example, metformin treatment alters the expression of SLCs transporting substrates ranging from Zn^2+^ and Cl^−^ to glycerol-3-phosphate and amino acids (Le et al., 2021), which underscores the metabolic impact of this drug. In fact, the significance of nutrient import in relation to cellular metabolic state is highly evolutionarily conserved. Unicellular organisms, such as yeasts and bacteria, also import non-essential metabolites, and different metabolic strategies may push cells to rely more on the extracellular metabolome. There is a clear logic behind this strategy: metabolite synthesis comes with the cost of resources, such as carbon, ATP or redox equivalents, including NAD^+^ or NADPH (reviewed previously by Keibler et al., 2016). Scavenging non-essential metabolites from the environment, therefore, increases the availability of metabolic precursors for alternative reactions (Barton et al., 2010). For example, respiratory-incompetent or NAD^+^-deficient *Saccharomyces cerevisiae* or *Schizosaccharomyces pombe*, despite being prototrophic in terms of amino acid production, show improved growth in medium supplemented with amino acids (Malecki et al., 2020; Vowinckel et al., 2021; Alam et al., 2023). Similarly, *S. cerevisiae* show increased tolerance to oxidative stress upon lysine supplementation, despite the fact that lysine is non-essential in the strains used. Lysine synthesis requires reduction, using NADPH at multiple steps, which diminishes the NADPH pool available for glutathione reductase, decreasing its capacity to remove reactive oxygen species (ROS) (D'Souza et al., 2014; Olin-Sandoval et al., 2019). *S. cerevisiae* was also shown to preferentially import metabolites if available and to downregulate the relevant biosynthetic pathways (Campbell et al., 2015). This underlines the significance of SLCs as main importers of extracellular metabolites that avoid costly ‘metabolic independence’.

Nutritional availability in different zones of a cell community, tissue or organ, inevitably leads to metabolic heterogeneity – even in a genetically homogenous community (Kondo et al., 2021). Even when cells are genetically capable of synthesising specific biomass components, if they lack the appropriate precursors to do so, they must rely on importing the final product through syntrophy (Box 1) (Andersen et al., 2015; Rodionova et al., 2015; Pacheco et al., 2019; Yu et al., 2022). A classic example of metabolic heterogeneity in a tissue is the retina: retinal pigmented epithelial (RPE) cells function as metabolic supporters for photoreceptors, which have a high turnover rate and energy demand but lack direct contact with the vasculature. Both RPE and photoreceptors rely on the expression of an array of SLCs to enable this supportive metabolic crosstalk. Recent work has shown that RPE cells preferentially take up proline and, therefore, have high expression levels of SLC6A20 (also known as SIT1, XT3 or XTRP3). RPE cells use this proline as an alternative nitrogen source for the synthesis of amino acids that are subsequently exported to photoreceptors in order to sustain their high metabolic rate (Takanaga et al., 2005; Liao et al., 2010; Strunnikova et al., 2010; Radeke et al., 2015; Chao et al., 2017; Liu et al., 2019; Li et al., 2020; Du et al., 2021).

Beyond the import of nutrients required for growth, SLCs allow cells to ‘sample’ nutrient availability: these extracellular metabolites can modulate intracellular metabolic signalling pathways, which enables cells to quickly adapt in response to changes in the extracellular metabolome (Hyde et al., 2007; Zhang et al., 2018; Pizzagalli et al., 2021). For example, reduced expression of the lactate transporter SLC16A1 (also known as MCT1) indirectly leads to AMPK activation (Carneiro et al., 2017), while inhibition of the plasma membrane amino acid transporter SLC7A5 (also known as LAT1) reduces mTOR activity (Imai et al., 2010; Ueno et al., 2016). SLCs on organellar membranes can also help regulate signalling pathways, impacting processes, such as protein synthesis (Tripathi et al., 2019). Even more directly, SLC transporters can take on a ‘transceptor’ role, i.e. that of a transporter and receptor (see reviews by Taylor, 2009; Kriel et al., 2011; Zheng et al., 2016; Pastor-Anglada and Pérez-Torras, 2018). For example, SLC38A9 (also known as URLC11) exports glutamine and/or arginine from the lysosome when lumen concentrations are high, such as in a fed state when cells are in amino acid-rich microenvironments. As well as exporting amino acids from lysosomal stores, the N-terminal domain of SLC38A9 provides a binding site for the Rag–Ragulator complex on the lysosome and promotes the activation of the Rag-GTPases (Shen and Sabatini, 2018). Its N-terminus has been shown to be exposed when SLC38A9 is transporting arginine, allowing activation of the Rag-GTPases and, in turn, of mTORC1 when arginine concentrations in the lysosome are high (Wu et al., 2006; Jung et al., 2015; Rebsamen et al., 2015; Wang et al., 2015b; Scalise et al., 2019; Lei et al., 2021). This positions SLC38A9 as a core sensory component of the mTORC1 regulatory machinery. Overall, SLC transporters play a key role in both sensing of and responding to the extracellular environment and are fundamental components of metabolic and signalling pathways.

## Transporters in cancer

Altered metabolism is a well-established hallmark of cancer, fuelling proliferation though the production of biosynthetic intermediates and maintenance of redox balance within a complex, often nutrient-depleted, microenvironment. Given the diversity of the SLC superfamily and their importance in shaping cellular metabolism, it is becoming increasingly appreciated that SLC transporters are central to this metabolic adaptation. As with their physiological roles, the contribution of SLCs to cancer are too numerous for us to comprehensively cover in this Review. Therefore, our aim is to cover the key thematic roles of transporters in cancer, illustrated with examples. Where available, we refer readers to additional publications for further reading.

### Accumulation of biosynthetic intermediates to support proliferation

Dividing cancer cells require exogenous carbon sources to generate ATP, biosynthetic intermediates and reducing power. Glucose is a major carbon source for cancer cells, and increased expression of SLC2 glucose transporter (GLUT) family members is among the best-known adaptations, as central contributors to the Warburg effect or aerobic glycolysis (Warburg, 1925; Warburg et al, 1927). In humans, SLC2A1 was the first of 14 GLUT family members to be identified and has been the most extensively studied to date (Kasahara and Hinkle, 1977; Carruthers et al., 2009; Thorens and Mueckler, 2010). A number of oncogenic and environmental alterations are associated with enhanced SLC2A1 expression in cancer, including mutation of EGFR or KRAS mutation, amplification of c-Myc and stabilisation of HIF1A (Box 1) (Ancey et al, 2018).

While metabolic changes in cancer are now known to be far more complex and heterogenous than can be encompassed by the Warburg hypothesis alone, the upregulation of SLC2A1 is a highly conserved feature of many tumour types (Brown and Wahl, 1993; Haber et al., 1998; Koh et al, 2017) and associated with decreased overall survival of cancer patients (Yu et al., 2017). Importantly, however, the enhanced glucose transport via GLUTs has been exploited for diagnostic and tumour-monitoring purposes with the development of FDG-PET imaging (Box 1) (Guerrero et al., 1990; Kelloff et al., 2005).

Increased glycolytic rates also lead to excess production of lactate, which can be transported into and out of the cell by the SLC16 family of monocarboxylate transporters. The highest capacity lactate exporters are SLC16A1 and SLC16A3 (MCT4), and their upregulation prevents intracellular acidosis.

Interestingly, lactate can be a carbon source, entering cells via SLC16A1, and the contribution of lactate to TCA cycle intermediates exceeds that of glucose in some tumour types (Hui et al., 2017). This adds to work by Sonveaux et al., who proposed a metabolic symbiosis model in some tumours, whereby glycolytic cancer cells export lactate via SLC16A3, which is subsequently imported via SLC16A1 for oxidative metabolism by cells within better-vascularised regions (Sonveaux et al., 2008). Furthermore, non-glucose carbon sources, including lactate, can be used even in well-perfused areas of non-small cell lung cancer patient tumours (Hensley et al., 2016).

In addition to lactate, monocarboxylate transporter substrates include pyruvate and ketone bodies, all of which are co-transported with a proton, rendering them sensitive to pH gradients across the membrane (Halestrap, 2013). Relative to SLC16A1 and SLC16A3, SLC16A7 (also known as MCT2) has been far less well studied in the context of cancer. However, SLC16A7 has the highest affinity for tested substrates of all members of the SLC16 family (Bröer et al., 1999), and a number of studies have demonstrated its increased expression in cancer, most notably in prostate carcinoma (Pértega-Gomes et al., 2012), where it has been suggested to be a putative biomarker. In breast cancer-derived lung metastases, SLC16A7 appears to be particularly influential as it drives cellular uptake of pyruvate. Downstream metabolism of this pyruvate promotes both the maintenance of mTORC1 signalling through increased activity of PHGDH (Rinaldi et al., 2021) and remodelling of the metastatic niche through hydroxylation of collagen (Elia et al., 2019). The increased production of α-ketoglutarate that results from pyruvate uptake has been implicated in driving these effects. As well as pyruvate, the uptake of adipocyte-derived β-hydroxybutyrate via SLC16A7 has been suggested to be pro-tumourigenic in breast cancer, as it has been linked to increased histone acetylation in the promotor region of tumour-promoting genes (Huang et al., 2017).

These studies highlight the importance of both metabolic and transporter plasticity to promote survival in dynamic nutrient states. As a result of their key roles in cancer cells, targeting GLUTs, MCTs and many other SLC transporters has been of major interest in recent years, with significant public and private funding deployed to improve understanding of this family (Wiedmer et al., 2022), as well as the development of several small-molecule inhibitors and clinical trials (Kopitz et al., 2016; Olszewski et al., 2022; Halford et al., 2023, see Table 1).

**Table 1.**
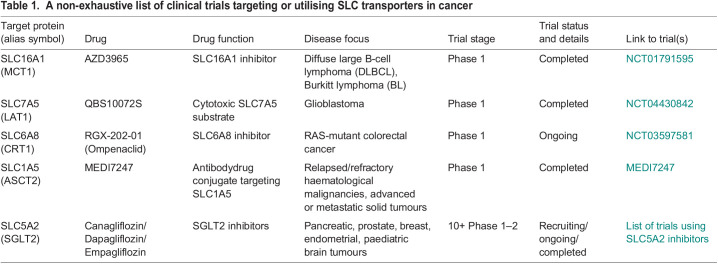
A non-exhaustive list of clinical trials targeting or utilising SLC transporters in cancer

Exogenous amino acids provide an essential nitrogen source and contribute to energy production, redox homeostasis and cell signalling. Glutamine, the most abundant amino acid in human plasma, is considered conditionally essential in the context of cancer, since cancer cells are often glutamine dependent (Wei et al., 2021; Yang et al., 2016).

High uptake rates of glutamine, as well as several other amino acids, are facilitated by multiple SLC families, including SLC1, which consists of two transport systems: the excitatory amino-acid-transporters SLC1A1, SLC1A2 and SLC1A3 (EAAT3, EAAT2 and EAAT1, respectively) and SLC1A6 and SLC1A7 (EAAT4 and EAAT5, respectively) and the alanine-serine-cysteine transporters SLC1A4 and SLC1A5 (ASCT1 and ASCT2, respectively). Notably, SLC1A5 is a Na^+^-dependent neutral amino acid exchanger, whose expression and/or activity is upregulated in response to the oncogenic drivers c-Myc (Wise et al., 2008; Gao et al., 2009) and EGFR (Avissar et al., 2008; Lu et al., 2016; Tao et al., 2017), and has been correlated with a significantly poorer outcome in patients. Silencing of SLC1A5 was shown to reduce growth in breast (van Geldermalsen et al., 2016), lung (Hassanein et al., 2013) and prostate cancer (Wang et al., 2015a), as well as acute myeloid leukaemia (Willems et al., 2013) and, subsequently, there has been a push to identify specific inhibitors for this transporter (Schulte et al., 2016). While successful in inhibiting cancer cell growth in *in vitro* and *in vivo* preclinical models of colorectal cancer (Schulte et al., 2018), further work has suggested that the mechanism of action of some of these inhibitors is through the targeting of two other glutamine transporters, SLC38A2 (SNAT2) and SLC7A5 (Bröer et al., 2019). Amino acids are directly sensed by mTORC1 and, as such, their transport into cells not only provides biosynthetic intermediates but also drives signal transduction pathways that stimulate cell growth. SLC7A5, an amino acid antiporter (Singh and Ecker, 2018), promotes the maintenance of essential amino acid levels, often in exchange for glutamine. The subsequent promotion of mTORC1 signalling demonstrates the importance of SLC7A5 in driving proliferation of KRAS-driven colorectal cancer, both *in vitro* and *in vivo* (Najumudeen et al., 2021).

### Transporter-mediated regulation of redox homeostasis

Altered cancer metabolism often leads to elevated levels of ROS, which can contribute to enhanced cancer progression and aggressiveness (Cheung and Vousden, 2022). ROS levels must, therefore, be kept in check, since uncontrolled elevation of ROS can damage DNA, proteins and lipids. Maintaining redox homeostasis is, therefore, critical for cancer cell survival. The cystine/glutamate antiporter SLC7A11 (also known as xCT) is a crucial regulator of redox homeostasis that functions by importing cystine into the cytoplasm in exchange for glutamate in a Na^+^-independent manner (Fig. 1). Cystine is the oxidised form of cysteine, a rate-limiting substrate for the biosynthesis of the antioxidant glutathione. While vital for maintaining redox balance, cystine is relatively insoluble, therefore, when import rates are high, it must be rapidly reduced to cysteine. This reduction reaction requires NADPH, which puts pressure on the pentose phosphate pathway (Box 1) (Liu et al., 2020). The subsequent increased dependence on glucose demonstrates that metabolic adaptation can often be coupled to novel vulnerabilities. Similarly, SLC7A11-driven depletion of the intracellular glutamate pool from cells that express high levels of this antiporter has also been demonstrated to increase sensitivity to glutamine deprivation in these cells (Ji et al., 2018).

Glutathione is a co-factor of glutathione peroxidase 4, an enzyme that eliminates toxic lipid peroxides, the build-up of which can induce ferroptosis (Box 1). Thus, SLC7A11 also promotes tumour survival through inhibition of ferroptosis (Badgley et al., 2020). Inhibition of SLC7A11 may, therefore, damage cancer cells by causing fatal ROS-mediated damage, making it an attractive pharmacological target.

### Transporter-driven tumourigenicity

As well as contributing to the metabolic rewiring of cancer cells to provide a growth or survival advantage, expression of some transporters or their isoforms has been suggested to directly contribute toward the tumourigenic potential of cells.

The mitochondrial pyruvate carrier (MPC), whose molecular identity remained elusive for many years, was identified by two groups in 2012 (Bricker et al., 2012; Herzig et al., 2012) and shown to be conserved from yeast to mammals. It is a heterodimeric complex formed of MPC1 and MPC2 (also known as SLC54A1 and SLC54A2, respectively) within the inner mitochondrial membrane (see Tavoulari et al., 2019; and the recent review by Tavoulari et al., 2023). By regulating the transport of pyruvate into the mitochondria, MPC sits at the interface between glycolysis and glucose oxidation, directly influencing the relative rates of each pathway. In several cancer types, decreased MPC expression correlates with increased aerobic glycolysis and reduced mitochondrial respiratory capacity (Li et al., 2017; Koh et al., 2018). In models of colorectal cancer, however, downregulation of MPC was sufficient to impair mitochondrial pyruvate oxidation. This promoted a glycolytic phenotype and the subsequent initiation of intestinal tumours both in mouse and fly models (Bensard et al., 2019). Supporting these studies, several reports have correlated reduced expression of the MPC with more aggressive cancer phenotypes and poorer patient prognosis (Schell et al., 2014; Zhong et al., 2015; Li et al., 2016; Wang et al., 2016). In contrast, within cholangiocarcinoma cells, increased *MPC1* (SLC54A1) expression driven by the transcriptional coactivator PGC1α (officially known as PPARGC1A) enhanced pyruvate entry into the mitochondria. This facilitated an increase in oxidative phosphorylation at the expense of aerobic glycolysis and a subsequent rise of ROS, which was associated with increased metastatic potential of xenografted cholangioacarcinoma cells (Li et al., 2018). Similarly, pharmacological inhibition of the MPC in androgen-receptor-driven prostate cancer models reduced proliferation of cancer cells *in vitro* and in xenografts (Bader et al., 2019), suggesting that the tumourigenic effect of MPC depends on the metabolic context of the cell it is expressed in.

The mitochondrial glutamine importer was recently identified to be a variant of the above-discussed SLC1A5 and, thus, named SLC1A5_var. SLC1A5_var is directed to the mitochondria through an N-terminal targeting sequence, where it promotes glutamine anaplerosis (Box 1) to fuel the TCA cycle and generate ATP, as well as increased glutathione synthesis to contribute to the maintenance of redox balance (Yoo et al., 2019). The identification of SLC1A5_var sheds further light on the capacity of metabolic reprogramming to drive tumourigenicity.

### Transporters as regulators of microenvironmental crosstalk

The tumour microenvironment is complex and dynamic, with cancer cells co-existing alongside a variety of immune cells, cancer-associated fibroblasts and endothelial cells, often perfused by a poorly formed vasculature. Among various modes of crosstalk within this environment, metabolite exchange has been shown to be highly important and to have implications not only for proliferation and survival but also drug resistance. Transporters are, of course, central to this exchange.

Pancreatic cancer has a particularly dense stromal architecture that causes poor vascular perfusion, resulting in a nutrient-depleted environment. Pancreatic stellate cells (PSCs), however, provide tumour cells with non-essential amino acids derived from cancer cell-stimulated autophagic breakdown of proteins within PSCs (Sousa et al., 2016). In particular, alanine taken up by the cancer cells is used to fuel the TCA cycle. This exchange is mediated by specific transporters; SLC1A4 facilitating non-essential amino acid efflux from PSCs and upregulated SLC38A2 enabling their uptake into the cancer cells (Parker et al., 2020).

Metabolic crosstalk can also modulate immune cells. Local depletion of methionine that results from SLC43A2 upregulation in cancer cells impairs CD8^+^ T-cell function (Bian et al., 2020). By contrast, metabolic rewiring induced by the uptake of tumour-cell-derived lactate inhibits succinate secretion by T-cells, which promotes autocrine signalling and tumour cell cytotoxicity (Elia et al., 2022). While this study did not characterise specific transporters, they no doubt constitute a central node of this cancer-immune system metabolic interaction.

Transporter-driven metabolic interactions can extend beyond the local environment and impact whole-body metabolic homeostasis. For example, in fly models that had been fed a high-sugar diet, FGF signalling from malignant tumours in the eye epithelium promoted systemic muscle wasting (Newton et al., 2020). The subsequent release of amino acids from muscle tissue, in particular proline, was capitalised on by upregulation of an SLC36 family orthologue to drive tumour growth (Newton et al., 2020).

### Metabolic heterogeneity

As with so many aspects of cancer, metabolism is heterogenous both at inter- and intra-tumoural level (Evers et al., 2019; Kim and DeBerardinis, 2019). It is clear that transporters play an important role in generating this metabolic heterogeneity. The human genome encodes a diverse range of SLC transporters (Fig. 2A). While the SLC and ABC transporter signatures in patient tumour samples largely cluster by tissue-of-origin (Fig. 2B), tumour samples express a far more varied complement of transporters than the corresponding normal tissue samples. This demonstrates substantial heterogeneity in transporter expression across tumours of the same type, probably originating from a combination of genetic variation in, e.g. oncogenic driver mutations, and non-genetic variations, such as proximity to blood vessels and subsequent nutritional availability. Given the established roles of transporters in cancer metabolism and their potential involvement in therapeutic response (see below), this heterogeneity could have significant implications for patients receiving cancer treatment.

## Transporters in drug uptake and efflux

As well as regulating endogenous metabolite transfer across lipid membranes, several families of transporters are better known for transporting exogenous substrates, affecting drug absorption, distribution, metabolism and excretion (ADME).

### Established drug transporter families

Whilst this article focuses on SLCs, the significance of ABC transporters in ADME warrants a brief discussion. The ABC family of transporters comprises 48 members (Moore et al., 2022) that actively use ATP hydrolysis to pump their substrates against their concentration gradient out of cells. Several, such as ABCB1 (also known as MDR1, P-glycoprotein), ABCG2 (also known as BCRP) or ABCC1 (also known as MRP1), have been widely studied due to their involvement in drug export and subsequent drug resistance. ABCB1, the best-characterized member of this family, has been associated with the transport of ∼200 compounds, including chemotherapeutic drugs, such as paclitaxel, irinotecan and olaparib (Vaidyanathan et al., 2016; César-Razquin et al., 2018). Given the number of ABCB1 interactions, the possibility of inhibiting the activity of this transporter has been explored to improve therapeutic efficacy of its substrates. Unfortunately, though three generations of ABCB1 inhibitors did improve the efficacy and toxicity profiles of chemotherapeutics during preclinical development, little success was seen in clinical trials (Robey et al., 2018).

Several SLC transporters from the SLCO (also known as OATP or SLC21), SLC22 (also known as OAT or OCT) and SLC47 (also known as MATE) families have also been well studied, particularly in the context of organs involved in drug absorption and metabolism, such as the intestine, liver and kidneys. Their activities, as well as those of a subset of ABC transporters, are recommended by the International Transporter Consortium to be investigated as part of the development, approval and safe use of all small-molecule drugs (Giacomini et al., 2018).

### A role for other SLC families?

**Fig. 3.**
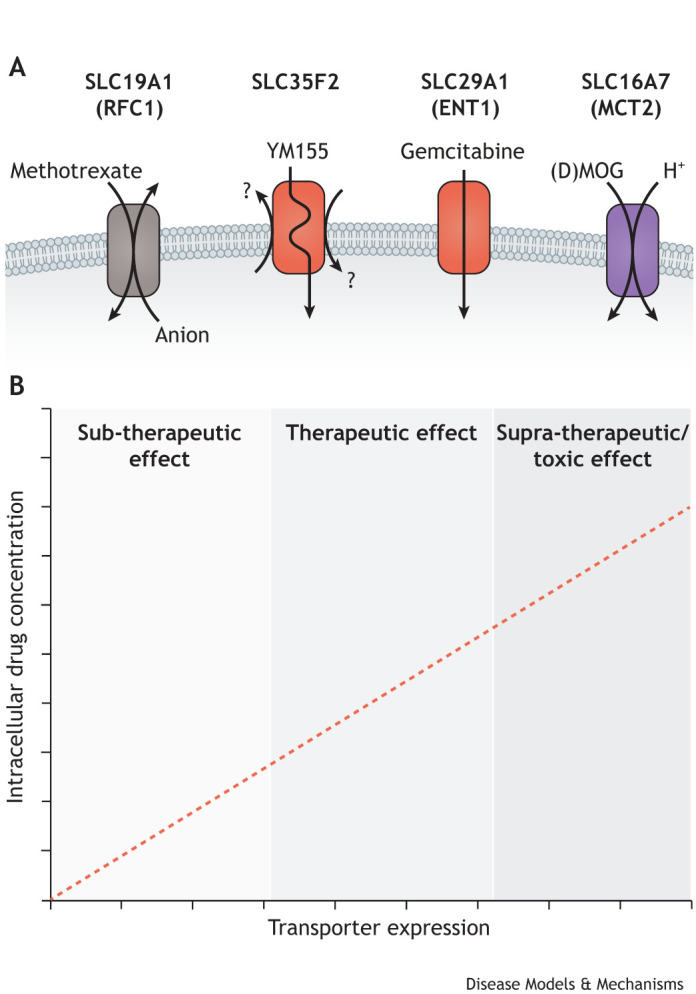
**Implications of transporter-dependent drug uptake for drug efficacy.** (A) Examples of SLC-dependent drug entry into target cells. A broad range of SLCs facilitate the entry of a chemically diverse spectrum of compounds. Members of the SLC21, SLC22 and SLC47 families are well-known drug transporters. Beyond these, several emerging SLCs, including those illustrated, are becoming recognised for their capacity to import drugs. The latter range from anti-metabolites, such as methotrexate (Murakami and Mori, 2012) and gemcitabine, to small-molecule inhibitors of metabolic enzymes, exemplified by MOG, a rapidly formed degradation product of the prolylhydroxylase inhibitor DMOG, and enzyme-interacting proteins, such as the survivin inhibitor YM155. The transporters are colour-coded based on their main substrate (see legend to Fig. 2 for details). (B) The intracellular concentration of a drug depends on the expression level of the transporter, which affects target engagement and, consequently, the therapeutic efficacy of the drug.

The role of SLC families beyond SLCO, SLC22 and SLC47 in the uptake of exogenous small molecules is less well understood. However, a number of studies have identified additional SLC transporters involved in drug uptake (Fig. 3A). Indeed, the chemical space occupied by many therapeutic compounds overlaps with that of endogenous metabolites (O'Hagan and Kell, 2017, 2020). Given that these transporters have evolved to regulate the transport of endogenous metabolites as well as exogenous natural products obtained from the diet, a broader role for transporters in drug uptake and efficacy warrants investigation. Expression of SLC16A1, for example, was shown to determine sensitivity to the glycolytic inhibitor 3-bromopyruvate (Birsoy et al., 2013; Skaripa-Koukelli et al., 2021), which is a candidate anti-cancer drug. Similarly, another monocarboxylate transporter, SLC16A7 (MCT2) facilitates the cellular uptake of MOG, leading to selective toxicity of DMOG (Box 1) in SLC16A7-expressing cells (Fets et al., 2018, 2022), which has implications for the use of this tool compound to inhibit prolyl hydroxylases. Several drugs, such as the chemotherapeutic melphalan (Begleiter et al., 1979), the key Parkinson's disease drug L-DOPA (Kageyama et al., 2000; Chien et al., 2018), the anticonvulsant gabapentin and its structural relative pregabalin (Dickens et al., 2013; Takahashi et al., 2018), are transported by SLC7A5. Sensitivity to the survivin inhibitor YM155, has also been shown to depend on its transporter SLC35F2, an orphan nucleoside transporter (Winter et al., 2014). YM155 has undergone numerous clinical trials in various cancers, with limited success (Li et al., 2019). It remains to be seen whether patient outcomes could be improved through stratification by SLC35F2 expression status.

### What proportion of drugs enter cells via transporters?

Classically, most small-molecule drugs were thought to diffuse through the lipid bilayer of plasma membranes dependent on their physicochemical properties, with predictions of relative permeability being approximated by ‘Lipinski's rule of five’ (Box 1) (Lipinski et al., 1997). While the above examples clearly illustrate that membrane transporters can play a role in drug uptake (Birsoy et al., 2013; Winter et al., 2014; Fets et al., 2018; Girardi et al., 2020), the mechanism of entry into target cells is not well characterised for the majority of drugs, and the proportion of drugs that require a transporter for cell entry has been extensively debated (Kell and Oliver, 2014; Smith et al., 2014; Cocucci et al., 2017; Kell, 2021).

Estimating the fraction of drugs that enter cells via transporters relative to those passing directly through the membrane requires a systematic approach. Previous large-scale studies have used gene knockout libraries or CRISPR screens to determine how the disruption of individual transporter genes shifts drug sensitivity. One study used the yeast gene knockout collection to demonstrate that presence of transporters is associated with sensitivity to 18 of the 26 tested compounds (Lanthaler et al., 2011). Using the haploid cell line HAP1, an SLC-specific CRISPR knockout library targeting 394 human SLC genes tested the transporter-dependent effects of 60 different drugs. 101 SLC transporters were associated with sensitivity to 47 of the 60 drugs (Girardi et al., 2020). This screening approach was validated by treating the model with the aforementioned survivin inhibitor YM155, demonstrating that expression of SLC35F2 is required for cell sensitivity to the drug; therefore, surviving cells were enriched for guide RNAs targeting this transporter (Winter et al., 2014).

These efforts suggested that the efficacy of at least 70–80% of tested compounds is influenced by the presence of transporters, with their loss leading to increased drug resistance. However, since many of these transporters have essential roles in cellular metabolism, it is possible that metabolic changes in cells lacking specific transporters affect their survival or drug sensitivity in a manner that is independent of compound uptake (Barnes et al., 2020). By contrast, gene knock-out approaches combined with cytotoxicity screening, like those described above, are unable to fully account for potential redundancy in drug uptake between different transporters (Evers et al., 2018) and could, therefore, lead researchers to underestimate their importance (Jindal et al., 2019). The uptake of metformin, for example, has been shown to be mediated by four different transporters in Caco-2 cell lines (Box 1) (Han et al., 2015).

### Importance of the metabolic context of transporters in drug uptake

Transport of the antifolate gemcitabine (Box 1) has been associated with several SLCs from both the SLC28 (CNT) family of concentrative nucleoside transporters and the SLC29 (ENT) family of equilibrative nucleoside transporters. Expression of SLC29A1 (also known as ENT1) has been shown to determine the sensitivity of cancer cell lines to gemcitabine (Mackey et al., 1998; García-Manteiga et al., 2003). This indicates that transporter expression levels could have real clinical impact (Farrell et al., 2009). Perhaps unsurprisingly, given the number of potential transporters for gemcitabine, some studies have reported inconsistent results, highlighting the need for a better understanding of gemcitabine uptake (Kawada et al., 2012; Mohelnikova-Duchonova and Melichar, 2013; Carter et al., 2021).

Interestingly, however, pancreatic cancer-associated macrophages secrete pyrimidine nucleosides. Given that gemcitabine enters cancer cells via nucleoside transporters of the SLC28 and SLC29 family, this microenvironment-derived competition for the transporter decreases drug uptake and was shown to lead to drug resistance in pre-clinical models (Halbrook et al., 2019). In the case of transporter-dependent drugs, therefore, the metabolic context of the tumour and the presence or absence of competitive endogenous substrates can be crucial to determining the uptake of a compound and, consequently, its therapeutic efficacy.

However, by understanding the modes of regulation of transporters involved in uptake or efflux of therapeutic compounds, it may be possible to manipulate their expression or activity through pharmacological or dietary intervention to maximise efficacy of the drug in question. This concept has been demonstrated in a proof-of-principle study, in which a small drug-fragment library was screened in a pancreatic cancer cell line for capability to increase sensitivity to gemcitabine, identifying a small molecule that decreased expression of the efflux transporter ABCC2, rendering cells more sensitive to this chemotherapeutic agent (Grixti et al., 2017).

### The implications of transporter-mediated uptake on drug efficacy in cancer

Understanding how a drug enters its target cell is of major importance since it has implications for predicting efficacy. If a drug requires a transporter for entry into a cell, then the level of expression of that transporter will dictate the intracellular concentration that the compound is able to reach (Fig. 3B). Our own work has illustrated this through the identification of SLC16A7 as a transporter for MOG and its analogues (Fets et al., 2018, 2022). MOG chemically stabilises the transcription factor HIF1A by inhibiting prolyl hydroxylases. While MOG-mediated inhibition of prolyl hydroxylases occurred in all tested cell lines, in those with high SLC16A7 expression, the active compound reached sufficient levels to bind several other, lower affinity α-ketoglutarate-dependent enzymes, thereby disrupting glutamine metabolism and causing toxicity (Fets et al., 2018).

Although DMOG is a tool compound and not clinically utilised, our study demonstrates that intracellular concentration can determine the functionality of a compound – too low and intended targets will not be sufficiently engaged, too high and the drug will interfere with unintended targets. This supratherapeutic effect occurs because, at higher concentrations, many ‘specific’ compounds interact with other targets as they reach selectivity limits (Fig. 3B) (Karaman et al., 2008). This means that transporter-dependent uptake could have implications for many targeted therapies, from inhibitors of tyrosine kinases to PARPs (Box 1) to PI3-kinases (Karaman et al., 2008; Lannutti et al., 2011; Knezevic et al., 2016). Similarly, high transporter expression could explain tissue-selective drug accumulation and associated toxicity in non-target tissues (Pagliarusco et al., 2011). Although relevant for all therapeutic compounds, the high inter-patient variability of transporter expression in cancer (Fig. 2B) means that a better understanding of how chemotherapeutic drugs interact with transporters could drastically improve the way we treat patients. If many drugs are, indeed, taken up via membrane transporters, the transporter signature of the tumour of an individual patient could dictate which compounds are able to reach therapeutically relevant intracellular concentrations. Identification of drug–transporter interactions could, therefore, provide functional biomarkers for the efficacy of therapeutic compounds in patients, allow stratification for clinical trials and, potentially, help to understand toxicities in non-target tissues.

### Concluding remarks

**Fig. 4.**
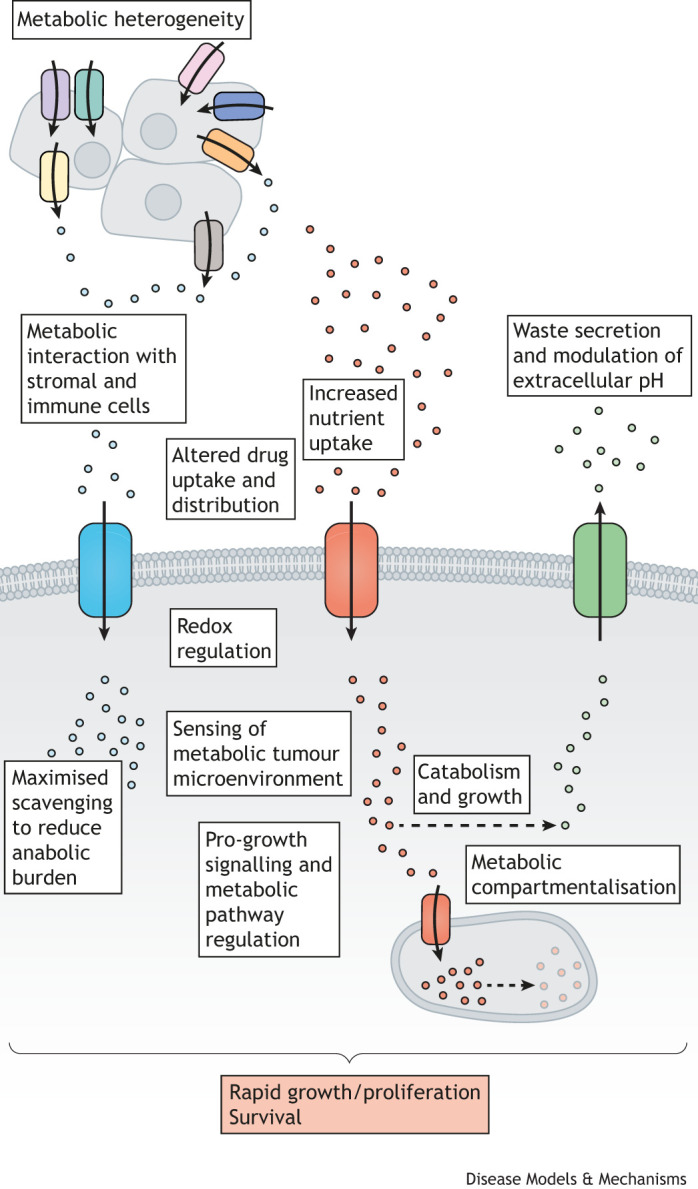
Infographic to illustrate how transporters are co-opted in cancer.

Though a relatively understudied group of proteins for many years (César-Razquin et al., 2015), what is already known about transporters and, particularly, SLCs, puts them at the heart of many physiological as well as disease processes (Fig. 4) and makes clear that more research is needed to understand their pharmacological impacts. Over recent years, the central role of transporters in many aspects of cancer has also been acknowledged through systematic screens using CRISPR libraries tailored to SLC and ABC transporter subfamilies, as demonstrated in recent publications (Li et al., 2021) and preprints (Chidley et al., 2023 preprint). These approaches and the tools they generate will expedite our understanding of metabolite transport in both physiological and disease states, particularly cancer, and fast-track the identification of novel SLC targets for therapeutic intervention.

It is clear, however, that transporter expression in cancer is heterogeneous, and this could have important pharmacological implications for many small-molecule therapeutics. In the era of personalised medicine, understanding the influence of transporters on drug import could enable us to develop bespoke treatment regimens based not only on mutations that drive the tumour of a patient, but also tailored to the unique transporter expression profile of that tumour. Though this Review has mainly focused on cancer, the principles of transporter-mediated drug uptake could, of course, be applicable to many therapeutic interventions and should, therefore, be considered as part of drug development and toxicity studies. While the expression of membrane transporters may not be as heterogeneous outside of the cancer space, their impact on drug efficacy though SNPs or drug-drug interactions are still highly relevant to a broad range of pharmacological agents.

## Supplementary Material

10.1242/dmm.050404_sup1 Supplementary information

Table S1.

## References

[DMM050404C1] Alam, M. T., Olin-Sandoval, V., Stincone, A., Keller, M. A., Zelezniak, A., Luisi, B. F. and Ralser, M. (2017). The self-inhibitory nature of metabolic networks and its alleviation through compartmentalization. *Nat. Commun.* 8, 16018. 10.1038/ncomms16018 28691704 PMC5508129

[DMM050404C2] Alam, S., Gu, Y., Reichert, P., Bähler, J. and Oliferenko, S. (2023). Optimization of energy production and central carbon metabolism in a non-respiring eukaryote. *Curr. Biol.* 33, 2175-2186.e5. 10.1016/j.cub.2023.04.046 37164017 PMC7615655

[DMM050404C3] Almannai, M., Alasmari, A., Alqasmi, A., Faqeih, E., Al Mutairi, F., Alotaibi, M., Samman, M. M., Eyaid, W., Aljadhai, Y. I., Shamseldin, H. E. et al. (2018). Expanding the phenotype of SLC25A42–associated mitochondrial encephalomyopathy. *Clin. Genet.* 93, 1097-1102. 10.1111/cge.13210 29327420

[DMM050404C4] Ancey, P., Contat, C. and Meylan, E. (2018). Glucose transporters in cancer – from tumor cells to the tumor microenvironment. *FEBS J.* 285, 2926-2943. 10.1111/febs.14577 29893496

[DMM050404C5] Andersen, S. B., Marvig, R. L., Molin, S., Krogh Johansen, H. and Griffin, A. S. (2015). Long-term social dynamics drive loss of function in pathogenic bacteria. *Proc. Natl Acad. Sci. USA* 112, 10756-10761. 10.1073/pnas.1508324112 26240352 PMC4553784

[DMM050404C6] Andreini, C., Bertini, I., Cavallaro, G., Holliday, G. L. and Thornton, J. M. (2009). Metal-MACiE: a database of metals involved in biological catalysis. *Bioinformatics* 25, 2088-2089. 10.1093/bioinformatics/btp256 19369503

[DMM050404C7] Aulakh, S. K., Varma, S. J. and Ralser, M. (2022). Metal ion availability and homeostasis as drivers of metabolic evolution and enzyme function. *Curr. Opin. Genet. Dev.* 77, 101987. 10.1016/j.gde.2022.101987 36183585

[DMM050404C8] Avissar, N. E., Sax, H. C. and Toia, L. (2008). In human entrocytes, GLN transport and ASCT2 Surface expression induced by short-term EGF are MAPK, PI3K, and Rho-dependent. *Dig. Dis. Sci.* 53, 2113-2125. 10.1007/s10620-007-0120-y 18157695

[DMM050404C9] Aykac, A. and Sehirli, A. O. (2020). The role of the SLC transporters protein in the neurodegenerative disorders. *Clin. Psychopharmacol. Neurosci.* 18, 174-187. 10.9758/cpn.2020.18.2.174 32329299 PMC7236796

[DMM050404C10] Bader, D. A., Hartig, S. M., Putluri, V., Foley, C., Hamilton, M. P., Smith, E. A., Saha, P. K., Panigrahi, A., Walker, C., Zong, L. et al. (2019). Mitochondrial pyruvate import is a metabolic vulnerability in androgen receptor-driven prostate cancer. *Nat. Metab.* 1, 70-85. 10.1038/s42255-018-0002-y 31198906 PMC6563330

[DMM050404C11] Badgley, M. A., Kremer, D. M., Maurer, H. C., Delgiorno, K. E., Lee, H.-J., Purohit, V., Sagalovskiy, I. R., Ma, A., Kapilian, J., Firl, C. E. M. et al. (2020). Cysteine depletion induces pancreatic tumor ferroptosis in mice. *Science* 368, 85-89. 10.1126/science.aaw9872 32241947 PMC7681911

[DMM050404C12] Barnes, E. M. E., Xu, Y., Benito, A., Herendi, L., Siskos, A. P., Aboagye, E. O., Nijhuis, A. and Keun, H. C. (2020). Lactic acidosis induces resistance to the pan-Akt inhibitor uprosertib in colon cancer cells. *Br. J. Cancer* 122, 1298-1308. 10.1038/s41416-020-0777-y 32152504 PMC7188671

[DMM050404C13] Bar-Peled, L. and Kory, N. (2022). Principles and functions of metabolic compartmentalization. *Nat. Metab.* 4, 1232-1244. 10.1038/s42255-022-00645-2 36266543 PMC10155461

[DMM050404C14] Barragán-Álvarez, C. P., Padilla-Camberos, E., Díaz, N. F., Cota-Coronado, A., Hernández-Jiménez, C., Bravo-Reyna, C. C. and Díaz-Martínez, N. E. (2021). Loss of Znt8 function in diabetes mellitus: risk or benefit? *Mol. Cell. Biochem.* 476, 2703-2718. 10.1007/s11010-021-04114-4 33666829

[DMM050404C15] Barton, M. D., Delneri, D., Oliver, S. G., Rattray, M. and Bergman, C. M. (2010). Evolutionary systems biology of amino acid biosynthetic cost in yeast. *PLoS ONE* 5, e11935. 10.1371/journal.pone.0011935 20808905 PMC2923148

[DMM050404C16] Begleiter, A., Lam, H. Y., Grover, J., Froese, E. and Goldenberg, G. J. (1979). Evidence for active transport of melphalan by two amino acid carriers in L5178Y lymphoblasts in vitro. *Cancer Res.* 39, 353-359. 570091

[DMM050404C17] Bensard, C. L., Wisidagama, D. R., Olson, K. A., Berg, J. A., Krah, N. M., Schell, J. C., Nowinski, S. M., Fogarty, S., Bott, A. J., Wei, P. et al. (2019). Regulation of tumor initiation by the mitochondrial pyruvate carrier. *Cell Metab.* 31, 284-300.e7. 10.1016/j.cmet.2019.11.002 31813825 PMC7004878

[DMM050404C18] Bessman, S. P. and Geiger, P. J. (1981). Transport of energy in muscle: the phosphorylcreatine shuttle. *Science* 211, 448-452. 10.1126/science.6450446 6450446

[DMM050404C19] Bian, Y., Li, W., Kremer, D. M., Sajjakulnukit, P., Li, S., Crespo, J., Nwosu, Z. C., Zhang, L., Czerwonka, A., Pawłowska, A. et al. (2020). Cancer SLC43A2 alters T cell methionine metabolism and histone methylation. *Nature* 585, 277-282. 10.1038/s41586-020-2682-1 32879489 PMC7486248

[DMM050404C20] Birsoy, K., Wang, T., Possemato, R., Yilmaz, O. H., Koch, C. E., Chen, W. W., Hutchins, A. W., Gultekin, Y., Peterson, T. R., Carette, J. E. et al. (2013). MCT1-mediated transport of a toxic molecule is an effective strategy for targeting glycolytic tumors. *Nat. Genet.* 45, 104-108. 10.1038/ng.2471 23202129 PMC3530647

[DMM050404C21] Boedtkjer, E., Praetorius, J., Matchkov, V. V., Stankevicius, E., Mogensen, S., Füchtbauer, A. C., Simonsen, U., Füchtbauer, E.-M. and Aalkjaer, C. (2011). Disruption of Na+,HCO3− cotransporter NBCn1 (slc4a7) inhibits NO-mediated vasorelaxation, smooth muscle Ca2+ sensitivity, and hypertension development in mice. *Circulation* 124, 1819-1829. 10.1161/circulationaha.110.015974 21947296

[DMM050404C22] Bricker, D. K., Taylor, E. B., Schell, J. C., Orsak, T., Boutron, A., Chen, Y.-C., Cox, J. E., Cardon, C. M., Van Vranken, J. G., Dephoure, N. et al. (2012). A mitochondrial pyruvate carrier required for pyruvate uptake in yeast, drosophila, and humans. *Science* 337, 96-100. 10.1126/science.1218099 22628558 PMC3690818

[DMM050404C23] Bröer, A., Gauthier-Coles, G., Rahimi, F., Van Geldermalsen, M., Dorsch, D., Wegener, A., Holst, J. and Bröer, S. (2019). Ablation of the ASCT2 (SLC1A5) gene encoding a neutral amino acid transporter reveals transporter plasticity and redundancy in cancer cells. *J. Biol. Chem.* 294, 4012-4026. 10.1074/jbc.ra118.006378 30635397 PMC6422075

[DMM050404C24] Bröer, S., Bröer, A., Schneider, H.-P., Stegen, C., Halestrap, A. P. and Deitmer, J. W. (1999). Characterization of the high-affinity monocarboxylate transporter MCT2 in Xenopus laevis oocytes. *Biochem. J.* 341, 529. 10.1042/0264-6021:3410529 10417314 PMC1220388

[DMM050404C25] Brown, R. S. and Wahl, R. L. (1993). Overexpression of glut–1 glucose transporter in human breast cancer an immunohistochemical study. *Cancer* 72, 2979-2985. 10.1002/1097-0142(19931115)72:10<;2979::aid-cncr2820721020>3.0.co;2-x 8221565

[DMM050404C26] Campbell, K., Mülleder, M., Malmsheimer, S., Lawrence, N., Calvani, E., Miller-Fleming, L., Alam, M. T., Christen, S., Keller, M. A. and Ralser, M. (2015). Self-establishing communities enable cooperative metabolite exchange in a eukaryote. *eLife* 4, e09943. 10.7554/elife.09943 26499891 PMC4695387

[DMM050404C27] Carneiro, L., Asrih, M., Repond, C., Sempoux, C., Stehle, J.-C., Leloup, C., Jornayvaz, F. R. and Pellerin, L. (2017). AMPK activation caused by reduced liver lactate metabolism protects against hepatic steatosis in MCT1 haploinsufficient mice. *Mol. Metab.* 6, 1625-1633. 10.1016/j.molmet.2017.10.005 29092796 PMC5699913

[DMM050404C28] Carruthers, A., Dezutter, J., Ganguly, A. and Devaskar, S. U. (2009). Will the original glucose transporter isoform please stand up!. *Am. J. Physiol. Endocrinol. Metabol.* 297, E836-E848. 10.1152/ajpendo.00496.2009 PMC2763785 19690067

[DMM050404C29] Carter, C. J., Mekkawy, A. H. and Morris, D. L. (2021). Role of human nucleoside transporters in pancreatic cancer and chemoresistance. *World J. Gastroenterol.* 27, 6844-6860. 10.3748/wjg.v27.i40.6844 34790010 PMC8567477

[DMM050404C30] Cervera-Acedo, C., Lopez, M., Aguirre-Lamban, J., Santibañez, P., Garcia-Oguiza, A., Poch-Olive, M. L. and Dominguez-Garrido, E. (2015). A novel SLC6A8 mutation associated with motor dysfunction in a child exhibiting creatine transporter deficiency. *Hum. Genome Var.* 2, 15037. 10.1038/hgv.2015.37 27081545 PMC4785581

[DMM050404C31] César-Razquin, A., Snijder, B., Frappier-Brinton, T., Isserlin, R., Gyimesi, G., Bai, X., Reithmeier, R. A., Hepworth, D., Hediger, M. A., Edwards, A. M. et al. (2015). A call for systematic research on solute carriers. *Cell* 162, 478-487. 10.1016/j.cell.2015.07.022 26232220

[DMM050404C32] César-Razquin, A., Girardi, E., Yang, M., Brehme, M., Saez-Rodriguez, J. and Superti-Furga, G. (2018). In silico prioritization of transporter–drug relationships from drug sensitivity screens. *Front. Pharmacol.* 09, 1011. 10.3389/fphar.2018.01011 PMC6137680 30245630

[DMM050404C33] Chao, J. R., Knight, K., Engel, A. L., Jankowski, C., Wang, Y., Manson, M. A., Gu, H., Djukovic, D., Raftery, D., Hurley, J. B. et al. (2017). Human retinal pigment epithelial cells prefer proline as a nutrient and transport metabolic intermediates to the retinal side. *J. Biol. Chem.* 292, 12895-12905. 10.1074/jbc.m117.788422 28615447 PMC5546030

[DMM050404C34] Chedik, L., Bruyere, A. and Fardel, O. (2019). Interactions of organophosphorus pesticides with solute carrier (SLC) drug transporters. *Xenobiotica* 49, 363-374. 10.1080/00498254.2018.1442030 29448871

[DMM050404C35] Cheung, E. C. and Vousden, K. H. (2022). The role of ROS in tumour development and progression. *Nat. Rev. Cancer* 22, 280-297. 10.1038/s41568-021-00435-0 35102280

[DMM050404C36] Chidley, C., Darnell, A. M., Gaudio, B. L., Lien, E. C., Barbeau, A. M., Vander Heiden, M. G. and Sorger, P. K. (2023). A CRISPRi/a screening platform to study cellular nutrient transport in diverse microenvironments. *bioRxiv*, 2023.01.26.525375. 10.1101/2023.01.26.525375 PMC11098743 38605144

[DMM050404C37] Chien, H.-C., Colas, C., Finke, K., Springer, S., Stoner, L., Zur, A. A., Venteicher, B., Campbell, J., Hall, C., Flint, A. et al. (2018). Reevaluating the substrate specificity of the L–type amino acid transporter (LAT1). *J. Med. Chem.* 61, 7358-7373. 10.1021/acs.jmedchem.8b01007 30048132 PMC6668346

[DMM050404C38] Chou, F.-S. and Wang, P.-S. (2020). The SLC25A42 transcript is a biomarker for fetal reprogramming in response to placental insufficiency in preterm newborns under 32 weeks gestation—a pilot study. *Front. Pediatr.* 8, 459. 10.3389/fped.2020.00459 32984199 PMC7485381

[DMM050404C39] Cocucci, E., Kim, J. Y., Bai, Y. and Pabla, N. (2017). Role of passive diffusion, transporters, and membrane trafficking–mediated processes in cellular drug transport. *Clin. Pharmacol. Ther.* 101, 121-129. 10.1002/cpt.545 27804130

[DMM050404C236] Colas, C., Ung, P.M. and Schlessinger, A. (2016). SLC transporters: structure, function, and drug discovery. *MedChemComm.* 7, 1069-1081. 10.1039/C6MD00005C 27672436 PMC5034948

[DMM050404C40] Crane, R. K. (1960). Intestinal absorption of sugars. *Physiol. Rev.* 40, 789-825. 10.1152/physrev.1960.40.4.789 13696269

[DMM050404C41] Crocco, P., Hoxha, E., Dato, S., De Rango, F., Montesanto, A., Rose, G. and Passarino, G. (2018). Physical decline and survival in the elderly are affected by the genetic variability of amino acid transporter genes. *Aging (Albany NY)* 10, 658-673. 10.18632/aging.101420 29676995 PMC5940118

[DMM050404C43] Czuba, L. C., Hillgren, K. M. and Swaan, P. W. (2018). Post-translational modifications of transporters. *Pharmacol. Ther.* 192, 88-99. 10.1016/j.pharmthera.2018.06.013 29966598 PMC6263853

[DMM050404C44] da Silva, R. P., Nissim, I., Brosnan, M. E. and Brosnan, J. T. (2009). Creatine synthesis: hepatic metabolism of guanidinoacetate and creatine in the rat in vitro and in vivo. *Am. J. Physiol. Endocrinol. Metab.* 296, E256-E261. 10.1152/ajpendo.90547.2008 19017728 PMC2645018

[DMM050404C45] Dickens, D., Webb, S. D., Antonyuk, S., Giannoudis, A., Owen, A., Rädisch, S., Hasnain, S. S. and Pirmohamed, M. (2013). Transport of gabapentin by LAT1 (SLC7A5). *Biochem. Pharmacol.* 85, 1672-1683. 10.1016/j.bcp.2013.03.022 23567998

[DMM050404C46] D'Souza, G., Waschina, S., Pande, S., Bohl, K., Kaleta, C. and Kost, C. (2014). Less is more: selective advantages can explain the prevalent loss of biosynthetic genes in bacteria. *Evolution* 68, 2559-2570. 10.1111/evo.12468 24910088

[DMM050404C235] Drew, D. and Boudker, O. (2015). Shared Molecular Mechanisms of Membrane Transporters. *Annu. Rev. Biochem.* 85, 1-30. 10.1146/annurev-biochem-060815-014520 27023848

[DMM050404C47] Du, J., Zhu, S., Lim, R. R. and Chao, J. R. (2021). Proline metabolism and transport in retinal health and disease. *Amino Acids* 53, 1789-1806. 10.1007/s00726-021-02981-1 33871679 PMC8054134

[DMM050404C237] Dvorak, V. and Superti-Furga, G. (2023). Structural and functional annotation of solute carrier transporters: implication for drug discovery. *Expert. Opin. Drug Discov*. 10.1080/17460441.2023.2244760 37563933

[DMM050404C48] Elia, I., Rossi, M., Stegen, S., Broekaert, D., Doglioni, G., Van Gorsel, M., Boon, R., Escalona-Noguero, C., Torrekens, S., Verfaillie, C. et al. (2019). Breast cancer cells rely on environmental pyruvate to shape the metastatic niche. *Nature* 568, 1-5. 10.1038/s41586-019-0977-x PMC6451642 30814728

[DMM050404C49] Elia, I., Rowe, J. H., Johnson, S., Joshi, S., Notarangelo, G., Kurmi, K., Weiss, S., Freeman, G. J., Sharpe, A. H. and Haigis, M. C. (2022). Tumor cells dictate anti-tumor immune responses by altering pyruvate utilization and succinate signaling in CD8+ T cells. *Cell Metab.* 34, 1137-1150.e6. 10.1016/j.cmet.2022.06.008 35820416 PMC9357162

[DMM050404C50] Evers, R., Piquette-Miller, M., Polli, J. W., Russel, F. G. M., Sprowl, J. A., Tohyama, K., Ware, J. A., De Wildt, S. N., Xie, W. and Brouwer, K. L. R. (2018). Disease–associated changes in drug transporters may impact the pharmacokinetics and/or toxicity of drugs: a white paper from the international transporter consortium. *Clin. Pharmacol. Ther.* 104, 900-915. 10.1002/cpt.1115 29756222 PMC6424581

[DMM050404C51] Evers, T. M. J., Hochane, M., Tans, S. J., Heeren, R. M. A., Semrau, S., Nemes, P. and Mashaghi, A. (2019). Deciphering metabolic heterogeneity by single-cell analysis. *Anal. Chem.* 91, 13314-13323. 10.1021/acs.analchem.9b02410 31549807 PMC6922888

[DMM050404C52] Fang, X., Liu, Y., Xiao, W., Zhao, N., Zhu, C., Yu, D. and Zhao, Y. (2021). Prognostic SLC family genes promote cell proliferation, migration, and invasion in hepatocellular carcinoma. *Acta Biochim. Biophys. Sin.* 53, gmab076. 10.1093/abbs/gmab076 34128989

[DMM050404C53] Farrell, J. J., Elsaleh, H., Garcia, M., Lai, R., Ammar, A., Regine, W. F., Abrams, R., Benson, A. B., Macdonald, J., Cass, C. E. et al. (2009). Human equilibrative nucleoside transporter 1 levels predict response to gemcitabine in patients with pancreatic cancer. *Gastroenterology* 136, 187-195. 10.1053/j.gastro.2008.09.067 18992248

[DMM050404C54] Ferrada, E. and Superti-Furga, G. (2022). A structure and evolutionary-based classification of solute carriers. *iScience* 25, 105096. 10.1016/j.isci.2022.105096 36164651 PMC9508557

[DMM050404C55] Fets, L., Driscoll, P. C., Grimm, F., Jain, A., Nunes, P. M., Gounis, M., Doglioni, G., Papageorgiou, G., Ragan, T. J., Campos, S. et al. (2018). MCT2 mediates concentration-dependent inhibition of glutamine metabolism by MOG. *Nat. Chem. Biol.* 14, 1032-1042. 10.1038/s41589-018-0136-y 30297875 PMC6298574

[DMM050404C56] Fets, L., Bevan, N., Nunes, P. M., Campos, S., Dos Santos, M. S., Sherriff, E., Macrae, J. I., House, D. and Anastasiou, D. (2022). MOG analogues to explore the MCT2 pharmacophore, α-ketoglutarate biology and cellular effects of N-oxalylglycine. *Commun. Biol.* 5, 877. 10.1038/s42003-022-03805-y 36028752 PMC9418262

[DMM050404C57] Fiermonte, G., Paradies, E., Todisco, S., Marobbio, C. M. T. and Palmieri, F. (2009). A novel member of solute carrier family 25 (SLC25A42) is a transporter of coenzyme a and adenosine 3′,5′-Diphosphate in human mitochondria*. *J. Biol. Chem.* 284, 18152-18159. 10.1074/jbc.m109.014118 19429682 PMC2709381

[DMM050404C58] Fitzsimmons, L., Liu, L., Porwollik, S., Chakraborty, S., Desai, P., Tapscott, T., Henard, C., Mcclelland, M. and Vazquez-Torres, A. (2018). Zinc-dependent substrate-level phosphorylation powers Salmonella growth under nitrosative stress of the innate host response. *PLoS Pathog.* 14, e1007388. 10.1371/journal.ppat.1007388 30365536 PMC6221366

[DMM050404C59] Freemerman, A. J., Johnson, A. R., Sacks, G. N., Milner, J. J., Kirk, E. L., Troester, M. A., Macintyre, A. N., Goraksha-Hicks, P., Rathmell, J. C. and Makowski, L. (2014). Metabolic reprogramming of macrophages glucose transporter 1 (GLUT1)-mediated glucose metabolism drives a proinflammatory phenotype*. *J. Biol. Chem.* 289, 7884-7896. 10.1074/jbc.m113.522037 24492615 PMC3953299

[DMM050404C60] Fox, E. R., Young, J. H., Li, Y., Dreisbach, A. W., Keating, B. J., Musani, S. K., Liu, K., Morrison, A. C., Ganesh, S., Kutlar, A. et al. (2011). Association of genetic variation with systolic and diastolic blood pressure among African Americans: the Candidate Gene Association Resource study. *Hum. Mol. Genet.* 20, 2273-2284. 10.1093/hmg/ddr092 21378095 PMC3090190

[DMM050404C61] Fujii, S. and Beutler, E. (1985). High glucose concentrations partially release hexokinase from inhibition by glucose 6-phosphate. *Proc. Natl Acad. Sci. USA* 82, 1552-1554. 10.1073/pnas.82.5.1552 3856279 PMC397302

[DMM050404C62] Gao, P., Tchernyshyov, I., Chang, T.-C., Lee, Y.-S., Kita, K., Ochi, T., Zeller, K. I., De Marzo, A. M., Van Eyk, J. E., Mendell, J. T. et al. (2009). c-Myc suppression of miR-23a/b enhances mitochondrial glutaminase expression and glutamine metabolism. *Nature* 458, 762-765. 10.1038/nature07823 19219026 PMC2729443

[DMM050404C63] García-Manteiga, J., Molina-Arcas, M., Casado, F. J., Mazo, A. and Pastor-Anglada, M. (2003). Nucleoside transporter profiles in human pancreatic cancer cells: role of hCNT1 in 2’,2’-difluorodeoxycytidine- induced cytotoxicity. *Clin. Cancer Res.* 9, 5000-5008. 14581375

[DMM050404C64] Geng, X., Liu, L., Banes-Berceli, A., Yang, Z., Kang, P., Shen, J., Tsai, K.-J. and Liu, Z. (2018). Role of ZIP8 in regulating cell morphology and NF-κB/Snail2 signaling. *Metallomics* 10, 953-964. 10.1039/c8mt00079d 29927450

[DMM050404C65] Gerosa, L. and Sauer, U. (2011). Regulation and control of metabolic fluxes in microbes. *Curr. Opin. Biotechnol.* 22, 566-575. 10.1016/j.copbio.2011.04.016 21600757

[DMM050404C66] Giacomini, K. M., Galetin, A. and Huang, S. M. (2018). The International Transporter Consortium: Summarizing Advances in the Role of Transporters in Drug Development. *Clin. Pharmacol. Ther.* 104, 766-771. 10.1002/cpt.1224 30137696

[DMM050404C67] Giménez, I., Isenring, P. and Forbush, B. (2002). Spatially distributed alternative splice variants of the renal Na-K-Cl cotransporter exhibit dramatically different affinities for the transported ions*. *J. Biol. Chem.* 277, 8767-8770. 10.1074/jbc.c200021200 11815599

[DMM050404C68] Girardi, E., César-Razquin, A., Lindinger, S., Papakostas, K., Konecka, J., Hemmerich, J., Kickinger, S., Kartnig, F., Gürtl, B., Klavins, K. et al. (2020). A widespread role for SLC transmembrane transporters in resistance to cytotoxic drugs. *Nat. Chem. Biol.* 16, 469-478. 10.1038/s41589-020-0483-3 32152546 PMC7610918

[DMM050404C69] Gorczyca, L., Du, J., Bircsak, K. M., Wen, X., Vetrano, A. M. and Aleksunes, L. M. (2021). Low oxygen tension differentially regulates the expression of placental solute carriers and ABC transporters. *FEBS Lett.* 595, 811-827. 10.1002/1873-3468.13937 32978975 PMC7987846

[DMM050404C70] Grixti, J. M., O'hagan, S., Day, P. J. and Kell, D. B. (2017). Enhancing drug efficacy and therapeutic index through cheminformatics-based selection of small molecule binary weapons that improve transporter-mediated targeting: a cytotoxicity system based on gemcitabine. *Front. Pharmacol.* 8, 155. 10.3389/fphar.2017.00155 28396636 PMC5366350

[DMM050404C71] Guerrero, T. M., Hoffman, E. J., Dahlbom, M., Cutler, P. D., Hawkins, R. A. and Phelps, M. E. (1990). Characterization of a whole body imaging technique for PET. *IEEE Trans. Nucl. Sci.* 37, 676-680. 10.1109/23.106697

[DMM050404C72] Haber, R. S., Pontious, C., Kovalenko, I., Lapienyte, L., Dreyer, S., Lee, H.-J., Thurston, G., Zhang, Y., Lazarus, J., Sajjakulnukit, P. et al. (1998). GLUT1 glucose transporter expression in colorectal carcinoma. *Cancer* 83, 34-40. 10.1002/(sici)1097-0142(19980701)83:1<;34::aid-cncr5>3.0.co;2-e 9655290

[DMM050404C73] Halbrook, C. J., Pontious, C., Kovalenko, I., Lapienyte, L., Dreyer, S., Lee, H.-J., Thurston, G., Zhang, Y., Lazarus, J., Sajjakulnukit, P. et al. (2019). Macrophage-released pyrimidines inhibit gemcitabine therapy in pancreatic cancer. *Cell Metab.* 29, 1390-1399.e6. 10.1016/j.cmet.2019.02.001 30827862 PMC6602533

[DMM050404C74] Halestrap, A. P. (2013). The SLC16 gene family â€” Structure, role and regulation in health and disease. *Mol. Asp. Med.* 34, 337-349. 10.1016/j.mam.2012.05.003 23506875

[DMM050404C75] Halford, S., Veal, G. J., Wedge, S. R., Payne, G. S., Bacon, C. M., Sloan, P., Dragoni, I., Heinzmann, K., Potter, S., Salisbury, B. M. et al. (2023). A phase I dose-escalation study of AZD3965, an oral monocarboxylate transporter 1 inhibitor, in patients with advanced cancer. *Clin. Cancer Res.* 29, 1429-1439. 10.1158/1078-0432.ccr-22-2263 36652553 PMC7614436

[DMM050404C76] Han, R., Sun, W. and Zhang, H. (2021). Identification of a signature comprising 5 soluble carrier family genes to predict the recurrence of papillary thyroid carcinoma. *Technol. Cancer Res. Treat.* 20, 15330338211036314. 10.1177/15330338211036314 34590520 PMC8489750

[DMM050404C77] Han, T. K., Proctor, W. R., Costales, C. L., Cai, H., Everett, R. S. and Thakker, D. R. (2015). Four cation-selective transporters contribute to apical uptake and accumulation of metformin in caco-2 cell monolayers. *J. Pharmacol. Exp. Ther.* 352, 519-528. 10.1124/jpet.114.220350 25563903 PMC4352590

[DMM050404C78] Hassanein, M., Hoeksema, M. D., Shiota, M., Qian, J., Harris, B. K., Chen, H., Clark, J. E., Alborn, W. E., Eisenberg, R. and Massion, P. P. (2013). SLC1A5 mediates glutamine transport required for lung cancer cell growth and survival. *Clin. Cancer Res.* 19, 560-570. 10.1158/1078-0432.ccr-12-2334 23213057 PMC3697078

[DMM050404C79] Hediger, M. A., Romero, M. F., Peng, J.-B., Rolfs, A., Takanaga, H. and Bruford, E. A. (2004). The ABCs of solute carriers: physiological, pathological and therapeutic implications of human membrane transport proteins. *Pflügers Archiv* 447, 465-468. 10.1007/s00424-003-1192-y 14624363

[DMM050404C80] Hediger, M. A., Clémençon, B., Burrier, R. E. and Bruford, E. A. (2013). The ABCs of membrane transporters in health and disease (SLC series): Introduction. *Mol. Asp. Med.* 34, 95-107. 10.1016/j.mam.2012.12.009 PMC3853582 23506860

[DMM050404C81] Hensley, C. T., Faubert, B., Yuan, Q., Lev-Cohain, N., Jin, E., Kim, J., Jiang, L., Ko, B., Skelton, R., Loudat, L. et al. (2016). Metabolic heterogeneity in human lung tumors. *Cell* 164, 681-694. 10.1016/j.cell.2015.12.034 26853473 PMC4752889

[DMM050404C82] Herzig, S., Raemy, E., Montessuit, S., Veuthey, J.-L., Zamboni, N., Westermann, B., Kunji, E. R. S. and Martinou, J.-C. (2012). Identification and functional expression of the mitochondrial pyruvate carrier. *Science* 337, 93-96. 10.1126/science.1218530 22628554

[DMM050404C83] Hodges, W. T., Jarasvaraparn, C., Ferguson, D., Griffett, K., Gill, L. E., Chen, Y., Ilagan, M. X. G., Hegazy, L., Elgendy, B., Cho, K. et al. (2022). Mitochondrial pyruvate carrier inhibitors improve metabolic parameters in diet-induced obese mice. *J. Biol. Chem.* 298, 101554. 10.1016/j.jbc.2021.101554 34973337 PMC8808181

[DMM050404C84] Huang, C.-K., Chang, P.-H., Kuo, W.-H., Chen, C.-L., Jeng, Y.-M., Chang, K.-J., Shew, J.-Y., Hu, C.-M. and Lee, W.-H. (2017). Adipocytes promote malignant growth of breast tumours with monocarboxylate transporter 2 expression via-hydroxybutyrate. *Nat. Commun.* 8, 13. 10.1038/ncomms14706 28281525 PMC5353665

[DMM050404C85] Hui, S., Ghergurovich, J. M., Morscher, R. J., Jang, C., Teng, X., Lu, W., Esparza, L. A., Reya, T., Le Zhan, W.-H., Yanxiang Guo, J. et al. (2017). Glucose feeds the TCA cycle via circulating lactate. *Nature* 551, 115-118. 10.1038/nature24057 29045397 PMC5898814

[DMM050404C86] Hyde, R., Cwiklinski, E. L., Macaulay, K., Taylor, P. M. and Hundal, H. S. (2007). Distinct sensor pathways in the hierarchical control of SNAT2, a putative amino acid transceptor, by amino acid availability*. *J. Biol. Chem.* 282, 19788-19798. 10.1074/jbc.m611520200 17488712

[DMM050404C87] Imai, H., Kaira, K., Oriuchi, N., Shimizu, K., Tominaga, H., Yanagitani, N., Sunaga, N., Ishizuka, T., Nagamori, S., Promchan, K. et al. (2010). Inhibition of L-type amino acid transporter 1 has antitumor activity in non-small cell lung cancer. *Anticancer Res.* 30, 4819-4828. 21187458

[DMM050404C88] International Consortium for Blood Pressure Genome-Wide Association Studies (2011). Genetic variants in novel pathways influence blood pressure and cardiovascular disease risk. *Nature* 478, 103-109. 10.1038/nature10405 21909115 PMC3340926

[DMM050404C89] Ji, X., Qian, J., Rahman, S. M. J., Siska, P. J., Zou, Y., Harris, B. K., Hoeksema, M. D., Trenary, I. A., Heidi, C., Eisenberg, R. et al. (2018). xCT (SLC7A11)-mediated metabolic reprogramming promotes non-small cell lung cancer progression. *Oncogene* 37, 5007-5019. 10.1038/s41388-018-0307-z 29789716 PMC6127081

[DMM050404C90] Jindal, S., Yang, L., Day, P. J. and Kell, D. B. (2019). Involvement of multiple influx and efflux transporters in the accumulation of cationic fluorescent dyes by Escherichia coli. *BMC Microbiol.* 19, 195. 10.1186/s12866-019-1561-0 31438868 PMC6704527

[DMM050404C91] Jung, J., Genau, H. M. and Behrends, C. (2015). Amino acid-dependent mtorc1 regulation by the lysosomal membrane protein SLC38A9. *Mol. Cell. Biol.* 35, 2479-2494. 10.1128/mcb.00125-15 25963655 PMC4475919

[DMM050404C92] Kageyama, T., Nakamura, M., Matsuo, A., Yamasaki, Y., Takakura, Y., Hashida, M., Kanai, Y., Naito, M., Tsuruo, T., Minato, N. et al. (2000). The 4F2hc/LAT1 complex transports l-DOPA across the blood–brain barrier. *Brain Res.* 879, 115-121. 10.1016/s0006-8993(00)02758-x 11011012

[DMM050404C93] Kanai, Y., Lee, W. S., You, G., Brown, D. and Hediger, M. A. (1994). The human kidney low affinity Na+/glucose cotransporter SGLT2. Delineation of the major renal reabsorptive mechanism for D-glucose. *J. Clin. Investig.* 93, 397-404. 10.1172/jci116972 8282810 PMC293794

[DMM050404C94] Karaman, M. W., Herrgard, S., Treiber, D. K., Gallant, P., Atteridge, C. E., Campbell, B. T., Chan, K. W., Ciceri, P., Davis, M. I., Edeen, P. T. et al. (2008). A quantitative analysis of kinase inhibitor selectivity. *Nat. Biotechnol.* 26, 127-132. 10.1038/nbt1358 18183025

[DMM050404C95] Kasahara, M. and Hinkle, P. C. (1977). Reconstitution and purification of the D-glucose transporter from human erythrocytes. *J. Biol. Chem.* 252, 7384-7390. 10.1016/s0021-9258(19)66976-0 903365

[DMM050404C96] Kawada, N., Uehara, H., Katayama, K., Nakamura, S., Takahashi, H., Ohigashi, H., Ishikawa, O., Nagata, S. and Tomita, Y. (2012). Human equilibrative nucleoside transporter 1 level does not predict prognosis in pancreatic cancer patients treated with neoadjuvant chemoradiation including gemcitabine. *J. Hepatobiliary Pancreat. Sci.* 19, 717-722. 10.1007/s00534-012-0514-x 22426593

[DMM050404C97] Kazak, L. and Cohen, P. (2020). Creatine metabolism: energy homeostasis, immunity and cancer biology. *Nat. Rev. Endocrinol.* 16, 421-436. 10.1038/s41574-020-0365-5 32493980

[DMM050404C98] Keibler, M. A., Wasylenko, T. M., Kelleher, J. K., Iliopoulos, O., Vander Heiden, M. G. and Stephanopoulos, G. (2016). Metabolic requirements for cancer cell proliferation. *Cancer Metab.* 4, 16. 10.1186/s40170-016-0156-6 27540483 PMC4989334

[DMM050404C99] Kell, D. B. (2021). The transporter-mediated cellular uptake and efflux of pharmaceutical drugs and biotechnology products: how and why phospholipid bilayer transport is negligible in real biomembranes. *Molecules* 26, 5629. 10.3390/molecules26185629 34577099 PMC8470029

[DMM050404C100] Kell, D. B. and Oliver, S. G. (2014). How drugs get into cells: tested and testable predictions to help discriminate between transporter-mediated uptake and lipoidal bilayer diffusion. *Front. Pharmacol.* 5, 231. 10.3389/fphar.2014.00231 25400580 PMC4215795

[DMM050404C101] Kelloff, G. J., Hoffman, J. M., Johnson, B., Scher, H. I., Siegel, B. A., Cheng, E. Y., Cheson, B. D., O'shaughnessy, J., Guyton, K. Z., Mankoff, D. A. et al. (2005). Progress and promise of FDG-PET imaging for cancer patient management and oncologic drug development. *Clin. Cancer Res.* 11, 2785-2808. 10.1158/1078-0432.ccr-04-2626 15837727

[DMM050404C102] Kim, J. and Deberardinis, R. J. (2019). Mechanisms and Implications of Metabolic Heterogeneity in Cancer. *Cell Metab.* 30, 434-446. 10.1016/j.cmet.2019.08.013 31484055 PMC6730674

[DMM050404C103] Knezevic, C. E., Wright, G., Remsing Rix, L. L., Kim, W., Kuenzi, B. M., Luo, Y., Watters, J. M., Koomen, J. M., Haura, E. B., Monteiro, A. N. et al. (2016). Proteome-wide profiling of clinical PARP inhibitors reveals compound-specific secondary targets. *Cell Chem. Biol.* 23, 1490-1503. 10.1016/j.chembiol.2016.10.011 27866910 PMC5182133

[DMM050404C104] Koh, Y. W., Lee, S. J. and Park, S. Y. (2017). Differential expression and prognostic significance of GLUT1 according to histologic type of non-small-cell lung cancer and its association with volume-dependent parameters. *Lung Cancer* 104, 31-37. 10.1016/j.lungcan.2016.12.003 28212997

[DMM050404C105] Koh, E., Kim, Y. K., Shin, D. and Kim, K.-S. (2018). MPC1 is essential for PGC-1α-induced mitochondrial respiration and biogenesis. *Biochem. J.* 475, 1687-1699. 10.1042/bcj20170967 29669911

[DMM050404C106] Kondo, H., Ratcliffe, C. D. H., Hooper, S., Ellis, J., Macrae, J. I., Hennequart, M., Dunsby, C. W., Anderson, K. I. and Sahai, E. (2021). Single-cell resolved imaging reveals intra-tumor heterogeneity in glycolysis, transitions between metabolic states, and their regulatory mechanisms. *Cell Rep.* 34, 108750. 10.1016/j.celrep.2021.108750 33596424 PMC7900713

[DMM050404C107] Kopitz, C., Toschi, L., Algire, C., Héroult, M., Frisk, A.-L., Meyer, K., Schmitz, A., Lagkadinou, E., Petrul, H., Heisler, I. et al. (2016). Abstract 4746: Pharmacological characterization of BAY-876, a novel highly selective inhibitor of glucose transporter (GLUT)-1 in vitro and in vivo. *Cancer Res.* 76 Suppl. 14, 4746-4746. 10.1158/1538-7445.am2016-4746

[DMM050404C108] Kriel, J., Haesendonckx, S., Rubio-Texeira, M., Van Zeebroeck, G. and Thevelein, J. M. (2011). From transporter to transceptor: signaling from transporters provokes re–evaluation of complex trafficking and regulatory controls. *BioEssays* 33, 870-879. 10.1002/bies.201100100 21913212 PMC3258547

[DMM050404C109] Kristensen, A. S., Andersen, J., Jørgensen, T. N., Sørensen, L., Eriksen, J., Loland, C. J., Strømgaard, K. and Gether, U. (2011). SLC6 Neurotransmitter transporters: structure, function, and regulation. *Pharmacol. Rev.* 63, 585-640. 10.1124/pr.108.000869 21752877

[DMM050404C110] Kunji, E. R. S., Aleksandrova, A., King, M. S., Majd, H., Ashton, V. L., Cerson, E., Springett, R., Kibalchenko, M., Tavoulari, S., Crichton, P. G. et al. (2016). The transport mechanism of the mitochondrial ADP/ATP carrier. *Biochim. Biophys. Acta* 1863, 2379-2393. 10.1016/j.bbamcr.2016.03.015 27001633

[DMM050404C111] Lannutti, B. J., Meadows, S. A., Herman, S. E. M., Kashishian, A., Steiner, B., Johnson, A. J., Byrd, J. C., Tyner, J. W., Loriaux, M. M., Deininger, M. et al. (2011). CAL-101, a p110δ selective phosphatidylinositol-3-kinase inhibitor for the treatment of B-cell malignancies, inhibits PI3K signaling and cellular viability. *Blood* 117, 591-594. 10.1182/blood-2010-03-275305 20959606 PMC3694505

[DMM050404C112] Lanthaler, K., Bilsland, E., Dobson, P. D., Moss, H. J., Pir, P., Kell, D. B. and Oliver, S. G. (2011). Genome-wide assessment of the carriers involved in the cellular uptake of drugs: a model system in yeast. *BMC Biol.* 9, 70. 10.1186/1741-7007-9-70 22023736 PMC3280192

[DMM050404C113] Larsson, C., Påhlman, I. L. and Gustafsson, L. (2000). The importance of ATP as a regulator of glycolytic flux in Saccharomyces cerevisiae. *Yeast* 16, 797-809. 10.1002/1097-0061(20000630)16:9<;797::aid-yea553>3.0.co;2-5 10861904

[DMM050404C114] Le, J., Fu, Y., Han, Q., Wei, X., Ji, H., Chen, Y., Wang, Q., Pi, P., Li, J., Lin, X. et al. (2021). Restoration of mRNA expression of solute carrier proteins in liver of diet-induced obese mice by metformin. *Front. Endocrinol.* 12, 720784. 10.3389/fendo.2021.720784 PMC8515182 34659115

[DMM050404C115] Lei, H.-T., Mu, X., Hattne, J. and Gonen, T. (2021). A conformational change in the N terminus of SLC38A9 signals mTORC1 activation. *Structure* 29, 426-432.e8. 10.1016/j.str.2020.11.014 33296665 PMC9994763

[DMM050404C116] Leung, K. W., Gvritishvili, A., Liu, Y. and Tombran-Tink, J. (2012). ZIP2 and ZIP4 mediate age-related zinc fluxes across the retinal pigment epithelium. *J. Mol. Neurosci.* 46, 122-137. 10.1007/s12031-011-9536-0 21603979

[DMM050404C117] Lewis, S., Chen, L., Raghuram, V., Khundmiri, S. J., Chou, C.-L., Yang, C.-R. and Knepper, M. A. (2021). ‘SLC-omics’ of the kidney: solute transporters along the nephron. *Am. J. Physiol. Cell Physiol.* 321, C507-C518. 10.1152/ajpcell.00197.2021 34191628 PMC8461813

[DMM050404C118] Li, X., Gianoulis, T. A., Yip, K. Y., Gerstein, M. and Snyder, M. (2010). Extensive In Vivo metabolite-protein interactions revealed by large-scale systematic analyses. *Cell* 143, 639-650. 10.1016/j.cell.2010.09.048 21035178 PMC3005334

[DMM050404C119] Li, Y., Li, X., Kan, Q., Zhang, M., Li, X., Xu, R., Wang, J., Yu, D., Goscinski, M. A., Wen, J.-G. et al. (2016). Mitochondrial pyruvate carrier function is negatively linked to Warburg phenotype in vitro and malignant features in esophageal squamous cell carcinomas. *Oncotarget* 8, 1058-1073. 10.18632/oncotarget.13717 PMC5352034 27911865

[DMM050404C120] Li, X., Han, G., Li, X., Kan, Q., Fan, Z., Li, Y., Ji, Y., Zhao, J., Zhang, M., Grigalavicius, M. et al. (2017). Mitochondrial pyruvate carrier function determines cell stemness and metabolic reprogramming in cancer cells. *Oncotarget* 8, 46363-46380. 10.18632/oncotarget.18199 28624784 PMC5542273

[DMM050404C121] Li, D., Wang, C., Ma, P., Yu, Q., Gu, M., Dong, L., Jiang, W., Pan, S., Xie, C., Han, J. et al. (2018). PGC1α promotes cholangiocarcinoma metastasis by upregulating PDHA1 and MPC1 expression to reverse the Warburg effect. *Cell Death Dis.* 9, 466. 10.1038/s41419-018-0494-0 29700317 PMC5919932

[DMM050404C122] Li, F., Aljahdali, I. and Ling, X. (2019). Cancer therapeutics using survivin BIRC5 as a target: what can we do after over two decades of study? *J. Exp. Clin. Cancer Res.* 38, 368. 10.1186/s13046-019-1362-1 31439015 PMC6704566

[DMM050404C123] Li, B., Zhang, T., Liu, W., Wang, Y., Xu, R., Zeng, S., Zhang, R., Zhu, S., Gillies, M. C., Zhu, L. et al. (2020). Metabolic features of mouse and human retinas: rods versus cones, macula versus periphery. Retina versus RPE. *iScience* 23, 101672. 10.1016/j.isci.2020.101672 33196018 PMC7644940

[DMM050404C124] Li, K.-C., Girardi, E., Kartnig, F., Grosche, S., Pemovska, T., Bigenzahn, J. W., Goldmann, U., Sedlyarov, V., Bensimon, A., Schick, S. et al. (2021). Cell-surface SLC nucleoside transporters and purine levels modulate BRD4-dependent chromatin states. *Nat. Metab.* 3, 651-664. 10.1038/s42255-021-00386-8 33972798 PMC7612075

[DMM050404C125] Liao, J.-L., Yu, J., Huang, K., Hu, J., Diemer, T., Ma, Z., Dvash, T., Yang, X.-J., Travis, G. H., Williams, D. S. et al. (2010). Molecular signature of primary retinal pigment epithelium and stem-cell-derived RPE cells. *Hum. Mol. Genet.* 19, 4229-4238. 10.1093/hmg/ddq341 20709808 PMC3115666

[DMM050404C126] Lin, L., Yee, S. W., Kim, R. B. and Giacomini, K. M. (2015). SLC transporters as therapeutic targets: emerging opportunities. *Nat. Rev. Drug Discov.* 14, 543-560. 10.1038/nrd4626 26111766 PMC4698371

[DMM050404C127] Lindsley, J. E. and Rutter, J. (2006). Whence cometh the allosterome? *Proc. Natl Acad. Sci. USA* 103, 10533-10535. 10.1073/pnas.0604452103 16818878 PMC1502268

[DMM050404C128] Lipinski, C. A., Lombardo, F., Dominy, B. W. and Feeney, P. J. (1997). Experimental and computational approaches to estimate solubility and permeability in drug discovery and development settings. *Adv. Drug Delivery. Rev.* 23, 3-25. 10.1016/s0169-409x(96)00423-1 11259830

[DMM050404C129] Liu, M.-J., Bao, S., Gálvez-Peralta, M., Pyle, C. J., Rudawsky, A. C., Pavlovicz, R. E., Killilea, D. W., Li, C., Nebert, D. W., Wewers, M. D. et al. (2013). ZIP8 Regulates host defense through zinc-mediated inhibition of NF-κB. *Cell Rep.* 3, 386-400. 10.1016/j.celrep.2013.01.009 23403290 PMC3615478

[DMM050404C130] Liu, B., Calton, M. A., Abell, N. S., Benchorin, G., Gloudemans, M. J., Chen, M., Hu, J., Li, X., Balliu, B., Bok, D. et al. (2019). Genetic analyses of human fetal retinal pigment epithelium gene expression suggest ocular disease mechanisms. *Commun. Biol.* 2, 186. 10.1038/s42003-019-0430-6 31123710 PMC6527609

[DMM050404C131] Liu, X., Olszewski, K., Zhang, Y., Lim, E. W., Shi, J., Zhang, X., Zhang, J., Lee, H., Koppula, P., Lei, G. et al. (2020). Cystine transporter regulation of pentose phosphate pathway dependency and disulfide stress exposes a targetable metabolic vulnerability in cancer. *Nat. Cell Biol.* 22, 476-486. 10.1038/s41556-020-0496-x 32231310 PMC7194135

[DMM050404C132] Lu, H., Li, X., Lu, Y., Qiu, S. and Fan, Z. (2016). ASCT2 (SLC1A5) is an EGFR-associated protein that can be co-targeted by cetuximab to sensitize cancer cells to ROS-induced apoptosis. *Cancer Lett.* 381, 23-30. 10.1016/j.canlet.2016.07.020 27450723 PMC5017913

[DMM050404C133] Mackey, J. R., Mani, R. S., Selner, M., Mowles, D., Young, J. D., Belt, J. A., Crawford, C. R. and Cass, C. E. (1998). Functional nucleoside transporters are required for gemcitabine influx and manifestation of toxicity in cancer cell lines. *Cancer Res.* 58, 4349-4357. 9766663

[DMM050404C134] Macpherson, J. A. and Anastasiou, D. (2017). Allosteric regulation of metabolism in cancer: endogenous mechanisms and considerations for drug design. *Curr. Opin. Biotechnol.* 48, 102-110. 10.1016/j.copbio.2017.03.022 28431259

[DMM050404C135] Malecki, M., Kamrad, S., Ralser, M. and Bähler, J. (2020). Mitochondrial respiration is required to provide amino acids during fermentative proliferation of fission yeast. *EMBO Rep.* 21, e50845. 10.15252/embr.202050845 32896087 PMC7645267

[DMM050404C136] Mao, H., Sheng, J., Jia, J., Wang, C., Zhang, S., Li, H. and He, F. (2021). Aberrant SLC6A14 expression promotes proliferation and metastasis of colorectal cancer via enhancing the JAK2/STAT3 pathway. *Onco Targets Ther.* 14, 379-392. 10.2147/ott.s288709 33469314 PMC7812055

[DMM050404C137] Martinez, D. L., Tsuchiya, Y. and Gout, I. (2014). Coenzyme A biosynthetic machinery in mammalian cells. *Biochem. Soc. Trans.* 42, 1112-1117. 10.1042/bst20140124 25110011

[DMM050404C138] Meixner, E., Goldmann, U., Sedlyarov, V., Scorzoni, S., Rebsamen, M., Girardi, E. and Superti-Furga, G. (2020). A substrate–based ontology for human solute carriers. *Mol. Syst. Biol.* 16, e9652. 10.15252/msb.20209652 32697042 PMC7374931

[DMM050404C139] Menga, A., Palmieri, E. M., Cianciulli, A., Infantino, V., Mazzone, M., Scilimati, A., Palmieri, F., Castegna, A. and Iacobazzi, V. (2017). SLC25A26 overexpression impairs cell function via mtDNA hypermethylation and rewiring of methyl metabolism. *FEBS J.* 284, 967-984. 10.1111/febs.14028 28118529

[DMM050404C140] Mohelnikova-Duchonova, B. and Melichar, B. (2013). Human equilibrative nucleoside transporter 1 (hENT1): Do we really have a new predictive biomarker of chemotherapy outcome in pancreatic cancer patients? *Pancreatology* 13, 558-563. 10.1016/j.pan.2013.09.005 24280569

[DMM050404C141] Moore, J. M., Bell, E. L., Hughes, R. O. and Garfield, A. S. (2022). ABC transporters: human disease and pharmacotherapeutic potential. *Trends Mol. Med.* 29, 152-172. 10.1016/j.molmed.2022.11.001 36503994

[DMM050404C142] Morioka, S., Perry, J. S. A., Raymond, M. H., Medina, C. B., Zhu, Y., Zhao, L., Serbulea, V., Onengut-Gumuscu, S., Leitinger, N., Kucenas, S. et al. (2018). Efferocytosis induces a novel SLC program to promote glucose uptake and lactate release. *Nature* 563, 714-718. 10.1038/s41586-018-0735-5 30464343 PMC6331005

[DMM050404C143] Mount, D. B. (2014). Thick ascending limb of the loop of henle. *Clin. J. Am. Soc. Nephrol.* 9, 1974-1986. 10.2215/cjn.04480413 25318757 PMC4220766

[DMM050404C144] Murakami, T. and Mori, N. (2012). Involvement of multiple transporters-mediated transports in mizoribine and methotrexate pharmacokinetics. *Pharmaceuticals* 5, 802-836. 10.3390/ph5080802 24280676 PMC3763673

[DMM050404C145] Najumudeen, A. K., Ceteci, F., Fey, S. K., Hamm, G., Steven, R. T., Hall, H., Nikula, C. J., Dexter, A., Murta, T., Race, A. M. et al. (2021). The amino acid transporter SLC7A5 is required for efficient growth of KRAS-mutant colorectal cancer. *Nat. Genet.* 53, 16-26. 10.1038/s41588-020-00753-3 33414552

[DMM050404C146] Newton, H., Wang, Y.-F., Camplese, L., Mokochinski, J. B., Kramer, H. B., Brown, A. E. X., Fets, L. and Hirabayashi, S. (2020). Systemic muscle wasting and coordinated tumour response drive tumourigenesis. *Nat. Commun.* 11, 4653. 10.1038/s41467-020-18502-9 32938923 PMC7495438

[DMM050404C147] Nicklisch, S. C. T. and Hamdoun, A. (2020). Disruption of small molecule transporter systems by Transporter–Interfering Chemicals (TICs). *FEBS Lett.* 594, 4158-4185. 10.1002/1873-3468.14005 33222203 PMC8112642

[DMM050404C148] Nielsen, T. T. (1983). Plasma citrate in relation to glucose and free fatty acid metabolism in man. *Dan. Med. Bull.* 30, 357-378. 6227460

[DMM050404C149] Nimmanon, T., Ziliotto, S., Morris, S., Flanagan, L. and Taylor, K. M. (2017). Phosphorylation of zinc channel ZIP7 drives MAPK, PI3K and mTOR growth and proliferation signalling. *Metallomics* 9, 471-481. 10.1039/c6mt00286b 28205653 PMC5451890

[DMM050404C150] Nishimura, M. and Naito, S. (2008). Tissue-specific mRNA expression profiles of human solute carrier transporter superfamilies. *Drug Metab. Pharmacokinet* 23, 22-44. 10.2133/dmpk.23.22 18305372

[DMM050404C151] Nishito, Y. and Kambe, T. (2019). Zinc transporter 1 (ZNT1) expression on the cell surface is elaborately controlled by cellular zinc levels. *J. Biol. Chem.* 294, 15686-15697. 10.1074/jbc.ra119.010227 31471319 PMC6816103

[DMM050404C152] O'Hagan, S. and Kell, D. B. (2017). Consensus rank orderings of molecular fingerprints illustrate the most genuine similarities between marketed drugs and small endogenous human metabolites, but highlight exogenous natural products as the most important ‘natural’ drug transporter substrates. *ADMET and DMPK* 5, 85-125. 10.5599/admet.5.2.376

[DMM050404C153] O'Hagan, S. and Kell, D. B. (2020). Structural similarities between some common fluorophores used in biology, marketed drugs, endogenous metabolites, and natural products. *Mar. Drugs* 18, 582. 10.3390/md18110582 33238416 PMC7700180

[DMM050404C154] O'Hagan, S., Wright Muelas, M., Day, P. J., Lundberg, E. and Kell, D. B. (2018). GeneGini: assessment via the Gini coefficient of reference ‘Housekeeping’ genes and diverse human transporter expression profiles. *Cell Syst.* 6, 230-244.e1. 10.1016/j.cels.2018.01.003 29428416 PMC5840522

[DMM050404C155] Olin-Sandoval, V., Yu, J. S. L., Miller-Fleming, L., Alam, M. T., Kamrad, S., Correia-Melo, C., Haas, R., Segal, J., Peña Navarro, D. A., Herrera-Dominguez, L. et al. (2019). Lysine harvesting is an antioxidant strategy and triggers underground polyamine metabolism. *Nature* 572, 249-253. 10.1038/s41586-019-1442-6 31367038 PMC6774798

[DMM050404C156] Olszewski, K., Barsotti, A., Feng, X.-J., Momcilovic, M., Liu, K. G., Kim, J.-I., Morris, K., Lamarque, C., Gaffney, J., Yu, X. et al. (2022). Inhibition of glucose transport synergizes with chemical or genetic disruption of mitochondrial metabolism and suppresses TCA cycle-deficient tumors. *Cell Chem. Biol.* 29, 423-435.e10. 10.1016/j.chembiol.2021.10.007 34715056

[DMM050404C157] Pacheco, A. R., Moel, M. and Segrè, D. (2019). Costless metabolic secretions as drivers of interspecies interactions in microbial ecosystems. *Nat. Commun.* 10, 103. 10.1038/s41467-018-07946-9 30626871 PMC6327061

[DMM050404C158] Pagliarusco, S., Martinucci, S., Bordini, E., Miraglia, L., Cufari, D., Ferrari, L. and Pellegatti, M. (2011). Tissue distribution and characterization of drug-related material in rats and dogs after repeated oral administration of casopitant. *Drug Metab. Dispos.* 39, 283-293. 10.1124/dmd.110.035063 20978104

[DMM050404C159] Parker, S. J., Amendola, C. R., Hollinshead, K. E. R., Yu, Q., Yamamoto, K., Encarnación-Rosado, J., Rose, R. E., Larue, M. M., Sohn, A. S. W., Biancur, D. E. et al. (2020). Selective alanine transporter utilization creates a targetable metabolic niche in pancreatic cancer. *Cancer Discov.* 10, 1018-1037. 10.1158/2159-8290.cd-19-0959 32341021 PMC7334074

[DMM050404C160] Pastor-Anglada, M. and Pérez-Torras, S. (2018). Emerging roles of nucleoside transporters. *Front. Pharmacol.* 9, 606. 10.3389/fphar.2018.00606 29928232 PMC5997781

[DMM050404C161] Pértega-Gomes, N., Vizcaíno, J. R., Gouveia, C., Jerónimo, C., Henrique, R. M., Lopes, C. and Baltazar, F. (2012). Monocarboxylate transporter 2 (MCT2) as putative biomarker in prostate cancer. *Prostate* 73, 763-769. 10.1002/pros.22620 23192371

[DMM050404C162] Pizzagalli, M. D., Bensimon, A. and Superti-Furga, G. (2021). A guide to plasma membrane solute carrier proteins. *FEBS J.* 288, 2784-2835. 10.1111/febs.15531 32810346 PMC8246967

[DMM050404C163] Radeke, M. J., Radeke, C. M., Shih, Y.-H., Hu, J., Bok, D., Johnson, L. V. and Coffey, P. J. (2015). Restoration of mesenchymal retinal pigmented epithelial cells by TGFβ pathway inhibitors: implications for age-related macular degeneration. *Genome Med.* 7, 58. 10.1186/s13073-015-0183-x 26150894 PMC4491894

[DMM050404C164] Rebsamen, M., Pochini, L., Stasyk, T., de Araújo, M. E. G., Galluccio, M., Kandasamy, R. K., Snijder, B., Fauster, A., Rudashevskaya, E. L., Bruckner, M. et al. (2015). SLC38A9 is a component of the lysosomal amino acid sensing machinery that controls mTORC1. *Nature* 519, 477-481. 10.1038/nature14107 25561175 PMC4376665

[DMM050404C165] Rinaldi, G., Pranzini, E., Van Elsen, J., Broekaert, D., Funk, C. M., Planque, M., Doglioni, G., Altea-Manzano, P., Rossi, M., Geldhof, V. et al. (2021). In Vivo evidence for serine biosynthesis-defined sensitivity of lung metastasis, but not of primary breast tumors, to mTORC1 inhibition. *Mol. Cell* 81, 386-397.e7. 10.1016/j.molcel.2020.11.027 33340488 PMC9161668

[DMM050404C166] Robey, R. W., Pluchino, K. M., Hall, M. D., Fojo, A. T., Bates, S. E. and Gottesman, M. M. (2018). Revisiting the role of ABC transporters in multidrug-resistant cancer. *Nat. Rev. Cancer* 18, 452-464. 10.1038/s41568-018-0005-8 29643473 PMC6622180

[DMM050404C167] Rodionova, I. A., Li, X., Plymale, A. E., Motamedchaboki, K., Konopka, A. E., Romine, M. F., Fredrickson, J. K., Osterman, A. L. and Rodionov, D. A. (2015). B–vitamin biosynthesis and transport in Chloroflexi. *Environ. Microbiol. Rep.* 7, 204-210. 10.1111/1758-2229.12227 25345570

[DMM050404C168] Rothstein, J. D. (1996). Excitotoxicity hypothesis. *Neurology* 47, 19S-26S. 10.1212/wnl.47.4_suppl_2.19s 8858047

[DMM050404C169] Ruprecht, J. J., Hellawell, A. M., Harding, M., Crichton, P. G., Mccoy, A. J. and Kunji, E. R. S. (2014). Structures of yeast mitochondrial ADP/ATP carriers support a domain-based alternating-access transport mechanism. *Proc. Natl Acad. Sci. USA* 111, E426-E434. 10.1073/pnas.1320692111 24474793 PMC3910652

[DMM050404C170] Sander, T., Farke, N., Diehl, C., Kuntz, M., Glatter, T. and Link, H. (2019). Allosteric feedback inhibition enables robust amino acid biosynthesis in E. coli by enforcing enzyme overabundance. *Cell Syst.* 8, 66-75.e8. 10.1016/j.cels.2018.12.005 30638812 PMC6345581

[DMM050404C171] Santarelli, S., Namendorf, C., Anderzhanova, E., Gerlach, T., Bedenk, B., Kaltwasser, S., Wagner, K., Labermaier, C., Reichel, J., Drgonova, J. et al. (2015). The amino acid transporter SLC6A15 is a regulator of hippocampal neurochemistry and behavior. *J. Psychiatr. Res.* 68, 261-269. 10.1016/j.jpsychires.2015.07.012 26228428

[DMM050404C172] Saunders, N. R., Dziegielewska, K. M., Møllgård, K., Habgood, M. D., Wakefield, M. J., Lindsay, H., Stratzielle, N., Ghersi-Egea, J.-F. and Liddelow, S. A. (2015). Influx mechanisms in the embryonic and adult rat choroid plexus: a transcriptome study. *Front. Neurosci.* 9, 123. 10.3389/fnins.2015.00123 25972776 PMC4412010

[DMM050404C173] Scalise, M., Galluccio, M., Pochini, L., Cosco, J., Trotta, M., Rebsamen, M., Superti-Furga, G. and Indiveri, C. (2019). Insights into the transport side of the human SLC38A9 transceptor. *Biochim. Biophys. Acta Biomembr.* 1861, 1558-1567. 10.1016/j.bbamem.2019.07.006 31295473

[DMM050404C174] Schell, J. C., Olson, K. A., Jiang, L., Hawkins, A. J., Van Vranken, J. G., Xie, J., Egnatchik, R. A., Earl, E. G., Deberardinis, R. J. and Rutter, J. (2014). A role for the mitochondrial pyruvate carrier as a repressor of the warburg effect and colon cancer cell growth. *Mol. Cell* 56, 400-413. 10.1016/j.molcel.2014.09.026 25458841 PMC4268416

[DMM050404C175] Schiessl, I. M., Rosenauer, A., Kattler, V., Minuth, W. W., Oppermann, M. and Castrop, H. (2013). Dietary salt intake modulates differential splicing of the Na-K-2Cl cotransporter NKCC2. *Am. J. Physiol. Renal Physiol.* 305, F1139-F1148. 10.1152/ajprenal.00259.2013 23946287

[DMM050404C176] Schoels, M., Zhuang, M., Fahrner, A., Küchlin, S., Sagar, M., Franz, H., Schmitt, A., Walz, G. and Yakulov, T. A. (2021). Single-cell mRNA profiling reveals changes in solute carrier expression and suggests a metabolic switch during zebrafish pronephros development. *Am. J. Physiol. Renal Physiol.* 320, F826-F837. 10.1152/ajprenal.00610.2020 33749326

[DMM050404C177] Schulte, M. L., Khodadadi, A. B., Cuthbertson, M. L., Smith, J. A. and Manning, H. C. (2016). 2-Amino-4-bis(aryloxybenzyl)aminobutanoic acids: a novel scaffold for inhibition of ASCT2-mediated glutamine transport. *Bioorg. Med. Chem. Lett.* 26, 1044-1047. 10.1016/j.bmcl.2015.12.031 26750251 PMC4727990

[DMM050404C178] Schulte, M. L., Fu, A., Zhao, P., Li, J., Geng, L., Smith, S. T., Kondo, J., Coffey, R. J., Johnson, M. O., Rathmell, J. C. et al. (2018). Pharmacological blockade of ASCT2-dependent glutamine transport leads to antitumor efficacy in preclinical models. *Nat. Med.* 24, 194-202. 10.1038/nm.4464 29334372 PMC5803339

[DMM050404C179] Shamseldin, H. E., Smith, L. L., Kentab, A., Alkhalidi, H., Summers, B., Alsedairy, H., Xiong, Y., Gupta, V. A. and Alkuraya, F. S. (2016). Mutation of the mitochondrial carrier SLC25A42 causes a novel form of mitochondrial myopathy in humans. *Hum. Genet.* 135, 21-30. 10.1007/s00439-015-1608-8 26541337 PMC4900140

[DMM050404C180] Shen, K. and Sabatini, D. M. (2018). Ragulator and SLC38A9 activate the Rag GTPases through noncanonical GEF mechanisms. *Proc. Natl Acad. Sci. USA* 115, 9545-9550. 10.1073/pnas.1811727115 30181260 PMC6156610

[DMM050404C181] Shimokawa, N., Okada, J., Haglund, K., Dikic, I., Koibuchi, N. and Miura, M. (2002). Past-A, a novel proton-associated sugar transporter, regulates glucose homeostasis in the brain. *J. Neurosci.* 22, 9160-9165. 10.1523/jneurosci.22-21-09160.2002 12417639 PMC6758044

[DMM050404C182] Singh, N. and Ecker, G. F. (2018). Insights into the structure, function, and ligand discovery of the large neutral amino acid transporter 1, LAT1. *Int. J. Mol. Sci.* 19, 1278. 10.3390/ijms19051278 29695141 PMC5983779

[DMM050404C183] Skaripa-Koukelli, I., Hauton, D., Walsby-Tickle, J., Thomas, E., Owen, J., Lakshminarayanan, A., Able, S., Mccullagh, J., Carlisle, R. C. and Vallis, K. A. (2021). 3-Bromopyruvate-mediated MCT1-dependent metabolic perturbation sensitizes triple negative breast cancer cells to ionizing radiation. *Cancer Metab.* 9, 37. 10.1186/s40170-021-00273-6 34649623 PMC8515664

[DMM050404C184] Slepchenko, K. G., Holub, J. M. and Li, Y. V. (2018). Intracellular zinc increase affects phosphorylation state and subcellular localization of protein kinase C delta (δ). *Cell. Signal.* 44, 148-157. 10.1016/j.cellsig.2018.01.018 29414441

[DMM050404C185] Smith, D., Artursson, P., Avdeef, A., Di, L., Ecker, G. F., Faller, B., Houston, J. B., Kansy, M., Kerns, E. H., Krämer, S. D. et al. (2014). Passive lipoidal diffusion and carrier-mediated cell uptake are both important mechanisms of membrane permeation in drug disposition. *Mol. Pharm.* 11, 1727-1738. 10.1021/mp400713v 24724562

[DMM050404C186] Song, W., Li, D., Tao, L., Luo, Q. and Chen, L. (2020). Solute carrier transporters: the metabolic gatekeepers of immune cells. *Acta Pharm. Sin. B* 10, 61-78. 10.1016/j.apsb.2019.12.006 31993307 PMC6977534

[DMM050404C187] Sonveaux, P., Végran, F., Schroeder, T., Wergin, M. C., Verrax, J., Rabbani, Z. N., De Saedeleer, C. J., Kennedy, K. M., Diepart, C., Jordan, B. et al. (2008). Targeting lactate-fueled respiration selectively kills hypoxic tumor cells in mice. *J. Clin. Investig.* 118, 3930-3942. 10.1172/jci36843 19033663 PMC2582933

[DMM050404C188] Sousa, C. M., Biancur, D. E., Wang, X., Halbrook, C. J., Sherman, M. H., Zhang, L., Kremer, D., Hwang, R. F., Witkiewicz, A. K., Ying, H. et al. (2016). Pancreatic stellate cells support tumour metabolism through autophagic alanine secretion. *Nature* 536, 479-483. 10.1038/nature19084 27509858 PMC5228623

[DMM050404C189] Strunnikova, N. V., Maminishkis, A., Barb, J. J., Wang, F., Zhi, C., Sergeev, Y., Chen, W., Edwards, A. O., Stambolian, D., Abecasis, G. et al. (2010). Transcriptome analysis and molecular signature of human retinal pigment epithelium. *Hum. Mol. Genet.* 19, 2468-2486. 10.1093/hmg/ddq129 20360305 PMC2876890

[DMM050404C190] Takahashi, Y., Nishimura, T., Higuchi, K., Noguchi, S., Tega, Y., Kurosawa, T., Deguchi, Y. and Tomi, M. (2018). Transport of pregabalin Via L-type amino acid transporter 1 (SLC7A5) in human brain capillary endothelial cell line. *Pharm. Res.* 35, 246. 10.1007/s11095-018-2532-0 30374619 PMC6208607

[DMM050404C191] Takanaga, H., Mackenzie, B., Suzuki, Y. and Hediger, M. A. (2005). Identification of mammalian proline transporter SIT1 (SLC6A20) with characteristics of classical system Imino*. *J. Biol. Chem.* 280, 8974-8984. 10.1074/jbc.m413027200 15632147

[DMM050404C192] Tamaki, N., Ikeda, T. and Funatsuka, A. (1983). Zinc as activating cation for muscle glycolysis. *J. Nutr. Sci. Vitaminol.* 29, 655-662. 10.3177/jnsv.29.655 6327959

[DMM050404C193] Tao, X., Lu, Y., Qiu, S., Wang, Y., Qin, J. and Fan, Z. (2017). AP1G1 is involved in cetuximab-mediated downregulation of ASCT2-EGFR complex and sensitization of human head and neck squamous cell carcinoma cells to ROS-induced apoptosis. *Cancer Lett.* 408, 33-42. 10.1016/j.canlet.2017.08.012 28823958 PMC5628150

[DMM050404C194] Tavoulari, S., Thangaratnarajah, C., Mavridou, V., Harbour, M. E., Martinou, J. C. and Kunji, E. R. S. (2019). The yeast mitochondrial pyruvate carrier is a hetero–dimer in its functional state. *EMBO J.* 38, e100785. 10.15252/embj.2018100785 30979775 PMC6517818

[DMM050404C195] Tavoulari, S., Sichrovsky, M. and Kunji, E. R. S. (2023). Fifty years of the mitochondrial pyruvate carrier: New insights into its structure, function, and inhibition. *Acta Physiol.* 238, e14016. 10.1111/apha.14016 PMC10909473 37366179

[DMM050404C196] Taylor, P. M. (2009). Amino acid transporters: éminences grises of nutrient signalling mechanisms? *Biochem. Soc. Trans.* 37, 237-241. 10.1042/bst0370237 19143639

[DMM050404C197] Thompson, K., Majd, H., Dallabona, C., Reinson, K., King, M. S., Alston, C. L., He, L., Lodi, T., Jones, S. A., Fattal-Valevski, A. et al. (2016). Recurrent De Novo dominant mutations in SLC25A4 cause severe early-onset mitochondrial disease and loss of mitochondrial DNA copy number. *Am. J. Hum. Genet.* 99, 860-876. 10.1016/j.ajhg.2016.08.014 27693233 PMC5065686

[DMM050404C198] Thorens, B. and Mueckler, M. (2010). Glucose transporters in the 21st Century. *Am. J. Physiol. Endocrinol. Metab.* 298, E141-E145. 10.1152/ajpendo.00712.2009 20009031 PMC2822486

[DMM050404C199] Tise, C. G., Palma, M. J., Cusmano-Ozog, K. P. and Matalon, D. R. (2023). Creatine transporter deficiency presenting as failure to thrive: a case report of a novel SLC6A8 variant causing a treatable but likely underdiagnosed genetic disorder. *J. Investig. Med. High Impact Case Rep.* 11, 23247096231154440. 10.1177/23247096231154438 PMC9909053 36752093

[DMM050404C200] Tripathi, R., Hosseini, K., Arapi, V., Fredriksson, R. and Bagchi, S. (2019). SLC38A10 (SNAT10) is located in ER and golgi compartments and has a role in regulating nascent protein synthesis. *Int. J. Mol. Sci.* 20, 6265. 10.3390/ijms20246265 31842320 PMC6940841

[DMM050404C201] Tweedie, S., Braschi, B., Gray, K., Jones, T. E. M., Seal, R. L., Yates, B. and Bruford, E. A. (2020). Genenames.org: the HGNC and VGNC resources in 2021. *Nucleic Acids Res.* 49, D939-D946. 10.1093/nar/gkaa980 PMC7779007 33152070

[DMM050404C202] Ueno, S., Kimura, T., Yamaga, T., Kawada, A., Ochiai, T., Endou, H. and Sakurai, H. (2016). Metformin enhances anti-tumor effect of L-type amino acid transporter 1 (LAT1) inhibitor. *J. Pharmacol. Sci.* 131, 110-117. 10.1016/j.jphs.2016.04.021 27262901

[DMM050404C203] Vaidyanathan, A., Sawers, L., Gannon, A.-L., Chakravarty, P., Scott, A. L., Bray, S. E., Ferguson, M. J. and Smith, G. (2016). ABCB1 (MDR1) induction defines a common resistance mechanism in paclitaxel- and olaparib-resistant ovarian cancer cells. *Br. J. Cancer* 115, 431-441. 10.1038/bjc.2016.203 27415012 PMC4985349

[DMM050404C204] Van Geldermalsen, M., Wang, Q., Nagarajah, R., Marshall, A. D., Thoeng, A., Gao, D., Ritchie, W., Feng, Y., Bailey, C. G., Deng, N. et al. (2016). ASCT2/SLC1A5 controls glutamine uptake and tumour growth in triple-negative basal-like breast cancer. *Oncogene* 35, 3201-3208. 10.1038/onc.2015.381 26455325 PMC4914826

[DMM050404C205] Vowinckel, J., Hartl, J., Marx, H., Kerick, M., Runggatscher, K., Keller, M. A., Mülleder, M., Day, J., Weber, M., Rinnerthaler, M. et al. (2021). The metabolic growth limitations of petite cells lacking the mitochondrial genome. *Nat. Metab.* 3, 1521-1535. 10.1038/s42255-021-00477-6 34799698 PMC7612105

[DMM050404C206] Wang, Q., Hardie, R. A., Hoy, A. J., Van Geldermalsen, M., Gao, D., Fazli, L., Sadowski, M. C., Balaban, S., Schreuder, M., Nagarajah, R. et al. (2015a). Targeting ASCT2–mediated glutamine uptake blocks prostate cancer growth and tumour development. *J. Pathol.* 236, 278-289. 10.1002/path.4518 25693838 PMC4973854

[DMM050404C207] Wang, S., Tsun, Z.-Y., Wolfson, R. L., Shen, K., Wyant, G. A., Plovanich, M. E., Yuan, E. D., Jones, T. D., Chantranupong, L., Comb, W. et al. (2015b). Lysosomal amino acid transporter SLC38A9 signals arginine sufficiency to mTORC1. *Science* 347, 188-194. 10.1126/science.1257132 25567906 PMC4295826

[DMM050404C208] Wang, L., Xu, M., Qin, J., Lin, S.-C., Lee, H.-J., Tsai, S. Y. and Tsai, M.-J. (2016). MPC1, a key gene in cancer metabolism, is regulated by COUPTFII in human prostate cancer. *Oncotarget* 7, 14673-14683. 10.18632/oncotarget.7405 26895100 PMC4924743

[DMM050404C209] Warburg, O. (1925). über den Stoffwechsel der Carcinomzelle. *Klinische Wochenschrift* 4, 534-536. 10.1007/bf01726151

[DMM050404C210] Warburg, O., Wind, F. and Negelein, E. (1927). The metabolism of tumors in the body. *J. Gen. Physiol.* 8, 519-530. 10.1085/jgp.8.6.519 19872213 PMC2140820

[DMM050404C211] Wei, Z., Liu, X., Cheng, C., Yu, W. and Yi, P. (2021). Metabolism of amino acids in cancer. *Front. Cell Dev. Biol.* 8, 603837. 10.3389/fcell.2020.603837 33511116 PMC7835483

[DMM050404C212] Wiedmer, T., Ingles-Prieto, A., Goldmann, U., Steppan, C. M. and Superti-Furga, G. (2022). Accelerating SLC transporter research: streamlining knowledge and validated tools. *Clin. Pharmacol. Ther.* 112, 439-442. 10.1002/cpt.2639 35938294 PMC9540488

[DMM050404C213] Wieman, H. L., Wofford, J. A. and Rathmell, J. C. (2007). Cytokine stimulation promotes glucose uptake via phosphatidylinositol-3 Kinase/Akt regulation of glut1 activity and trafficking. *Mol. Biol. Cell* 18, 1437-1446. 10.1091/mbc.e06-07-0593 17301289 PMC1838986

[DMM050404C214] Willems, L., Jacque, N., Jacquel, A., Neveux, N., Trovati Maciel, T., Lambert, M., Schmitt, A., Poulain, L., Green, A. S., Uzunov, M. et al. (2013). Inhibiting glutamine uptake represents an attractive new strategy for treating acute myeloid leukemia. *Blood* 122, 3521-3532. 10.1182/blood-2013-03-493163 24014241 PMC3829119

[DMM050404C215] Williamson, M. K., Coombes, N., Juszczak, F., Athanasopoulos, M., Khan, M., Eykyn, T., Srenathan, U., Taams, L., Dias Zeidler, J., Da Poian, A. et al. (2018). Upregulation of glucose uptake and hexokinase activity of primary human CD4+ T cells in response to infection with HIV-1. *Viruses* 10, 114. 10.3390/v10030114 29518929 PMC5869507

[DMM050404C216] Winter, G. E., Radic, B., Mayor-Ruiz, C., Blomen, V. A., Trefzer, C., Kandasamy, R. K., Huber, K. V. M., Gridling, M., Chen, D., Klampfl, T. et al. (2014). The solute carrier SLC35F2 enables YM155-mediated DNA damage toxicity. *Nat. Chem. Biol.* 10, 768-773. 10.1038/nchembio.1590 25064833 PMC4913867

[DMM050404C217] Wise, D. R., Deberardinis, R. J., Mancuso, A., Sayed, N., Zhang, X.-Y., Pfeiffer, H. K., Nissim, I., Daikhin, E., Yudkoff, M., Mcmahon, S. B. et al. (2008). Myc regulates a transcriptional program that stimulates mitochondrial glutaminolysis and leads to glutamine addiction. *Proc. Natl Acad. Sci. USA* 105, 18782-18787. 10.1073/pnas.0810199105 19033189 PMC2596212

[DMM050404C218] Wojtal, K. A., Cee, A., Lang, S., Götze, O., Frühauf, H., Geier, A., Pastor-Anglada, M., Torres-Torronteras, J., Martã­, R., Fried, M. et al. (2014). Downregulation of duodenal SLC transporters and activation of proinflammatory signaling constitute the early response to high altitude in humans. *Am. J. Physiol. Gastrointest. Liver Physiol.* 307, G673-G688. 10.1152/ajpgi.00353.2013 24970780

[DMM050404C219] Wright, E. M., Loo, D. D. F. and Hirayama, B. A. (2011). Biology of human sodium glucose transporters. *Physiol. Rev.* 91, 733-794. 10.1152/physrev.00055.2009 21527736

[DMM050404C220] Wu, B., Ottow, K., Poulsen, P., Gaber, R. F., Albers, E. and Kielland-Brandt, M. C. (2006). Competitive intra- and extracellular nutrient sensing by the transporter homologue Ssy1p. *J. Cell Biol.* 173, 327-331. 10.1083/jcb.200602089 16651382 PMC2063833

[DMM050404C221] Xu, Y., Cao, B., Chen, Y. P., Ou, R. W., Wei, Q. Q., Yang, J., Zhao, B., Song, W. and Shang, H.-F. (2016). SLC1A2 rs3794087 are associated with susceptibility to Parkinson's disease, but not essential tremor, amyotrophic lateral sclerosis or multiple system atrophy in a Chinese population. *J. Neurol. Sci.* 365, 96-100. 10.1016/j.jns.2016.04.003 27206883

[DMM050404C222] Yamashita, S., Miyagi, C., Fukada, T., Kagara, N., Che, Y.-S. and Hirano, T. (2004). Zinc transporter LIVI controls epithelial-mesenchymal transition in zebrafish gastrula organizer. *Nature* 429, 298-302. 10.1038/nature02545 15129296

[DMM050404C223] Yang, Y., Gozen, O., Watkins, A., Lorenzini, I., Lepore, A., Gao, Y., Vidensky, S., Brennan, J., Poulsen, D., Won Park, J. et al. (2009). Presynaptic regulation of astroglial excitatory neurotransmitter transporter GLT1. *Neuron* 61, 880-894. 10.1016/j.neuron.2009.02.010 19323997 PMC2743171

[DMM050404C224] Yang, L., Venneti, S. and Nagrath, D. (2016). Glutaminolysis: a hallmark of cancer metabolism. *Annu. Rev. Biomed. Eng.* 19, 1-32. 10.1146/annurev-bioeng-071516-044546 28301735

[DMM050404C225] Yi, C. and Yu, A.-M. (2022). MicroRNAs in the regulation of solute carrier proteins behind xenobiotic and nutrient transport in cells. *Front. Mol. Biosci.* 9, 893846. 10.3389/fmolb.2022.893846 35755805 PMC9220936

[DMM050404C226] Yoo, H. C., Park, S. J., Nam, M., Kang, J., Kim, K., Yeo, J. H., Kim, J.-K., Heo, Y., Lee, H. S., Lee, M. Y. et al. (2019). A variant of SLC1A5 is a mitochondrial glutamine transporter for metabolic reprogramming in cancer cells. *Cell Metab.* 31, 267-283.e12. 10.1016/j.cmet.2019.11.020 31866442

[DMM050404C227] Yu, M., Yongzhi, H., Chen, S., Luo, X., Lin, Y., Zhou, Y., Jin, H., Hou, B., Deng, Y., Tu, L. et al. (2017). The prognostic value of GLUT1 in cancers: a systematic review and meta-analysis. *Oncotarget* 8, 43356-43367. 10.18632/oncotarget.17445 28498810 PMC5522151

[DMM050404C228] Yu, J. S. L., C. Correia-Melo, F. Zorrilla, L. Herrera-Dominguez, M. Y. Wu, J. Hartl, K. Campbell, S. Blasche, M. Kreidl, A.-S. Egger, et al. (2022). Microbial communities form rich extracellular metabolomes that foster metabolic interactions and promote drug tolerance. *Nat. Microbiol.* 7, 542-555. 10.1038/s41564-022-01072-5 35314781 PMC8975748

[DMM050404C229] Zecchin, A., Stapor, P. C., Goveia, J. and Carmeliet, P. (2015). Metabolic pathway compartmentalization: an underappreciated opportunity? *Curr. Opin. Biotechnol.* 34, 73-81. 10.1016/j.copbio.2014.11.022 25499800

[DMM050404C230] Zhang, Y., Zhang, Y., Sun, K., Meng, Z. and Liu, F. (2018). The SLC transporter in nutrient and metabolic sensing, regulation, and drug development. *J. Mol. Cell Biol.* 11, 1-13. 10.1093/jmcb/mjy052 PMC6359923 30239845

[DMM050404C231] Zhang, C., Sui, D., Zhang, T. and Hu, J. (2020). Molecular basis of zinc-dependent endocytosis of human ZIP4 transceptor. *Cell Rep.* 31, 107582. 10.1016/j.celrep.2020.107582 32348750 PMC7661102

[DMM050404C232] Zheng, L., Zhang, W., Zhou, Y., Li, F., Wei, H. and Peng, J. (2016). Recent advances in understanding amino acid sensing mechanisms that regulate mTORC1. *Int. J. Mol. Sci.* 17, 1636. 10.3390/ijms17101636 27690010 PMC5085669

[DMM050404C233] Zhong, Y., Li, X., Yu, D., Li, X., Li, Y., Long, Y., Yuan, Y., Ji, Z., Zhang, M., Wen, J.-G. et al. (2015). Application of mitochondrial pyruvate carrier blocker UK5099 creates metabolic reprogram and greater stem-like properties in LnCap prostate cancer cells in vitro. *Oncotarget* 6, 37758-37769. 10.18632/oncotarget.5386 26413751 PMC4741963

[DMM050404C234] Zhou, S. and Shu, Y. (2022). Transcriptional regulation of solute carrier (SLC) drug transporters. *Drug Metab. Dispos.* 50, DMD-MR-2021-000704. 10.1124/dmd.121.000704 PMC9488976 35644529

