# Supplementary material for: Membrane transporters in cell physiology, cancer metabolism and drug response
Source: Dis Model Mech. 2023 Dec 1;16(11):dmm050404. doi: 10.1242/dmm.050404 (PMC10695176; doi:10.1242/dmm.050404)
Supplement: Supplementary information [file dmm-16-050404-s1.pdf]

Table S1.

Available for download at
https://journals.biologists.com/dmm/article-lookup/doi/10.1242/dmm.050404#supplementary-data

Disease Models & Mechanisms • Supplementary information
